# The mTOR regulated RNA-binding protein LARP1 requires PABPC1 for guided mRNA interaction

**DOI:** 10.1093/nar/gkaa1189

**Published:** 2020-12-17

**Authors:** Ewan M Smith, Nour El Houda Benbahouche, Katherine Morris, Ania Wilczynska, Sarah Gillen, Tobias Schmidt, Hedda A Meijer, Rebekah Jukes-Jones, Kelvin Cain, Carolyn Jones, Mark Stoneley, Joseph A Waldron, Cameron Bell, Bruno D Fonseca, Sarah Blagden, Anne E Willis, Martin Bushell

**Affiliations:** Cancer Research UK Beatson Institute, Garscube Estate, Switchback Road, Bearsden, Glasgow G61 1BD, UK; Cancer Research UK Beatson Institute, Garscube Estate, Switchback Road, Bearsden, Glasgow G61 1BD, UK; MRC Toxicology Unit, University of Cambridge, Leicester LE1 9HN, UK; Cancer Research UK Beatson Institute, Garscube Estate, Switchback Road, Bearsden, Glasgow G61 1BD, UK; Institute of Cancer Sciences, University of Glasgow, Garscube Estate, Switchback Road, Bearsden, Glasgow G61 1QH, UK; Cancer Research UK Beatson Institute, Garscube Estate, Switchback Road, Bearsden, Glasgow G61 1BD, UK; Cancer Research UK Beatson Institute, Garscube Estate, Switchback Road, Bearsden, Glasgow G61 1BD, UK; Division of Cell and Developmental Biology, School of Life Sciences, University of Dundee, Dundee DD1 5EH, UK; MRC Toxicology Unit, University of Cambridge, Leicester LE1 9HN, UK; MRC Toxicology Unit, University of Cambridge, Leicester LE1 9HN, UK; MRC Toxicology Unit, University of Cambridge, Leicester LE1 9HN, UK; MRC Toxicology Unit, University of Cambridge, Leicester LE1 9HN, UK; Cancer Research UK Beatson Institute, Garscube Estate, Switchback Road, Bearsden, Glasgow G61 1BD, UK; Cancer Research UK Therapeutic Discovery Laboratories, London Bioscience Innovation Centre, 2 Royal College Street, London NW1 0NH, UK; PrimerGen Ltd, Viseu, Portugal; Department of Oncology, University of Oxford, Oxford, OX3 7LE, UK; MRC Toxicology Unit, University of Cambridge, Leicester LE1 9HN, UK; Cancer Research UK Beatson Institute, Garscube Estate, Switchback Road, Bearsden, Glasgow G61 1BD, UK; Institute of Cancer Sciences, University of Glasgow, Garscube Estate, Switchback Road, Bearsden, Glasgow G61 1QH, UK

## Abstract

The mammalian target of rapamycin (mTOR) is a critical regulator of cell growth, integrating multiple signalling cues and pathways. Key among the downstream activities of mTOR is the control of the protein synthesis machinery. This is achieved, in part, *via* the co-ordinated regulation of mRNAs that contain a terminal oligopyrimidine tract (TOP) at their 5′ends, although the mechanisms by which this occurs downstream of mTOR signalling are still unclear. We used RNA-binding protein (RBP) capture to identify changes in the protein-RNA interaction landscape following mTOR inhibition. Upon mTOR inhibition, the binding of LARP1 to a number of mRNAs, including TOP-containing mRNAs, increased. Importantly, non-TOP-containing mRNAs bound by LARP1 are in a translationally-repressed state, even under control conditions. The mRNA interactome of the LARP1-associated protein PABPC1 was found to have a high degree of overlap with that of LARP1 and our data show that PABPC1 is required for the association of LARP1 with its specific mRNA targets. Finally, we demonstrate that mRNAs, including those encoding proteins critical for cell growth and survival, are translationally repressed when bound by both LARP1 and PABPC1.

## INTRODUCTION

The mammalian target of rapamycin (mTOR) is the catalytic component of two multi-protein complexes known as mTORC1 and mTORC2 with differing activities dependent upon its interacting partners (1–8). mTORC1 and mTORC2 are activated by upstream growth signals and control the balance between catabolic and anabolic processes through the phosphorylation of distinct substrates (reviewed in (9,10)). mTORC1, in particular, plays a major role in the regulation of protein synthesis (reviewed in (11–14)). mTORC1 mediated regulation of protein synthesis is achieved via several mechanisms (12,13). This includes the control of cap-dependent translation through the phosphorylation of the 4E binding proteins (4E-BP1–3 (15,16)) to regulate eIF4F complex formation, the activation of ribosomal protein S6 kinases (S6Ks) (17), which phosphorylate several substrates involved in translation elongation including the ribosomal protein S6 (18–22), and the eukaryotic elongation factor 2 kinase (eEF2K), which in turn modulates elongation rates through the phosphorylation of the eukaryotic elongation factor 2 (eEF2) (23).

A major group of co-regulated growth-associated mRNAs controlled by mTORC1 are the terminal oligopyrimidine tract (TOP) mRNAs, which encode ribosomal proteins and translation factors (24–27). TOP transcripts are defined by a distinctive motif at the 5′end of these mRNAs, which start with a cytosine after the m^7^GTP cap structure, followed directly by an uninterrupted pyrimidine tract 4–14 nucleosides in length (28–30). This feature is the determinant that allows rapid and reversible control of the biosynthesis of the translational apparatus *via* mTORC1 signalling (31). Ribosome profiling approaches have revealed that a similar mTOR dependent translational regulation may also occur on mRNAs that contain a pyrimidine-rich translational element (PRTE) downstream of the cap in the 5′UTR (31,32). Both TOP and PRTE regulation occurs in a nutrient and growth factor-dependent manner, allowing for the highly energy consuming process of ribosome production to be downregulated when cellular amino acid or energy levels become limiting. TOP mRNAs are highly stable and abundant in mammalian cells and the control of their expression is believed to occur predominantly at the level of translation (26,33–34). Several candidate *trans*-acting proteins and micro-RNAs have been previously proposed to regulate TOP-containing mRNA translation (35–37). Recent studies have identified La-related protein 1 (LARP1) as a protein that binds to both the m^7^Gppp-C cap and terminal oligopyrimidine tract (38–42). There has been controversy in the literature regarding whether LARP1 is a negative or positive regulator of translation. An original study by Tcherkezian *et al.* (41) reported that LARP1 stimulated the translation of TOP mRNAs upon insulin treatment; however, recent studies have found that rather than activating their translation, LARP1 represses TOP mRNA translation (38–40,42–43). The mechanism by which LARP1 causes repression is still incompletely understood, but it has been demonstrated that LARP1 binds directly to the cap-structure and adjacent 5′TOP motif of TOP mRNAs, thus competing with eIF4F complex for accessibility to their 5′UTR (39).

Recent development of a novel class of inhibitors of mTOR (44–46) that directly target the active site of mTOR allows for the inhibition of both complexes. Our study encompasses inhibition of mTORC1 and mTORC2 since both have been implicated in controlling translation (11–14,17,47–50) and potentially regulate RNA binding protein ineractions. mTORC2 is regulated by growth factors through phosphatidylinositol 3-kinase (PI3K) (5,51–53) and activity also appears to be stimulated by interaction with the ribosome in a protein-synthesis independent manner (47,48). mTORC2 phosphorylates and regulates several kinases including AKT, PKC and SGK1 (4,6,51,54–58). The main known functional output of mTORC2 is in actin cytoskeleton organization (1,4,6).

In this study, we characterized mTOR-regulated reorganization of ribonucleo-protein complexes, to determine how these proteins were controlling translation and investigate their influence on the life cycle of mRNA. Herein we used Torin1, a specific pharma-logical inhibitor of mTOR (mTORC1 and mTORC2) (45,46,59) in conjunction with whole cell RNA-binding protein capture to identify mTOR regulated mRNA trans-acting factors. This work led to the identification of 22 RNA-binding proteins differentially binding to RNA following mTOR inhibition with LARP1 and TRIM25 increasing their binding. Furthermore we characterized the RNAs that are bound to LARP1 and how the binding changes upon mTOR inhibition. We examined the properties of these mRNAs with regard to translation and present data to show that PABPC1 interaction is required for LARP1 binding specificity.

## MATERIALS AND METHODS

### Chemicals and reagents

Unless stated otherwise, all chemicals were purchased from Sigma-Aldrich or ThermoFisher Scientific. Torin1 was purchased from Tocris Bioscience (catalogue # 4247). Protein A/G plus agarose was purchased from Santa Cruz Biotechnology (catalogue # SC-2003). RNasin Plus RNase Inhibitor was purchased from Promega (catalogue # N2615). The following antibodies were used: Rabbit IgG (Santa Cruz Biotechnology, catalogue # SC2027); AKT (P) Ser473 (Cell Signalling Technologies, catalogue # 4060); S6K (Cell Signalling Technologies, catalogue # 2708); S6K (P) Thr389 (Cell Signalling Technologies, catalogue # 9234); RPS6 (Cell Signalling Technologies, catalogue # 2217); RPS6 (P) Ser240/244 (Cell Signalling Technologies, catalogue # 2215); 4E-BP1 (P) Thr37/46 (Cell Signalling Technologies, catalogue # 9459); 4E-BP1 (P) Ser65 (Cell Signalling Technologies, catalogue # 9451); 4E-BP1 (Cell Signalling Technologies, catalogue # 9644); eIF4E (Novus Biologicals, catalogue # NBP1–0058833); eIF4G (Cell Signalling Technologies, catalogue # 2498); β-tubulin (Santa Cruz Biotechnologies, catalogue # SC-9104); GAPDH (Santa Cruz Biotechnologies, catalogue # SC-32233); PABPC1 (kind gift from Professor Simon Morley); hnRNP-Q (Novus Biologicals, catalogue # NBP1–07043); PSF (Santa Cruz Biotechnology, catalogue # SC-101137); PTBP1 (ThermoFisher Scientific, catalogue # 32-4800); LARP1 (Novus Biologicals, catalogue # NBP1–19128); PWP2 (Atlas Antibodies, catalogue # HPA024573); SERBP1 (Bethyl Labs, catalogue # A303-938A); TRIM25 (Abcam, catalogue # ab167154); RPS10 (GeneTex, catalogue # GTX101836).

### Cell culture and treatment

HeLa cells were cultured in 4.5 g/l glucose DMEM (GIBCO Invitrogen Life Technologies catalogue # 21969-035) supplemented with 2 mM l-glutamine (GIBCO Invitrogen Life Technologies catalogue # 25030032) and 10% (v/v) foetal bovine serum (GIBCO Invitrogen Life Technologies catalogue # 10270-106) in a humidified, 5% (v/v) CO_2_ incubator at 37°C in the absence of antibiotics. Cells were routinely mycoplasma tested and also underwent cell line authentication using the GenePrint 10 kit (Promega, catalogue # B9510) and data was analysed using Applied Biosystems Genemapper v4.1 software. Prior to drug treatment, medium was supplemented with an extra 2% (v/v) foetal bovine serum and 2 mM l-glutamine for 30 min. Treatments were performed by the addition of 200 nM Torin1 or equivalent volume of DMSO vehicle (control) for 60 min prior to cell lysis.

### Whole cell RNA-binding protein (RBP) capture using oligo(dT) affinity isolation

Nine 15cm plates of HeLa cells were seeded at 1.8 million cells per plate per treatment. Twenty-four hours post-plating, cells were treated for 1 h ± 200 nM Torin1 as above. Medium was then aspirated and the cells washed twice with 8 mL ice-cold PBS. Plates were irradiated at 150 mJ/cm^2^ on ice in a 254 nm UV oven. Cells were lysed directly by scraping on the plate by adding 2 ml of oligo(dT) lysis/binding buffer (20 mM Tris pH7.4, 500 mM LiCl, 0.5% (w/v) LiDS, 1 mM EDTA, 5 mM DTT and protease inhibitor tablet) at room temperature. Lysates for each condition were pooled and passed through a 21G needle 10 times, followed by incubation at room temperature for 10 min with end-over-end rotation. Lysates centrifuged briefly at room temperature. Meanwhile, 0.5 ml magnetic oligo(dT) beads per 2 ml lysate were equilibrated in 1 ml lysis buffer with end-over-end rotation at room temperature for 5 min. Using a magnetic rack, buffer was removed and beads resuspended in 1.95 ml lysate. Remaining lysate was used as input sample. Pull-downs were incubated at room temperate for 1 h 15 min mixing end-over-end on a rotating wheel. Using a magnetic rack, the non-bound fraction was removed and beads were then washed twice for 10 min with constant rotation in 950 μl of wash buffer I (20 mM Tris pH 7.4, 500 mM LiCl, 0.1% (w/v) LiDS, 1 mM EDTA, 5 mM DTT), centrifuged briefly and then twice for 5 min with constant rotation in 950 μl of wash buffer II (20 mM Tris pH 7.4, 500 mM LiCl, 1 mM EDTA), and centrifuged briefly again. Beads were resuspended gently 2–3 times in 0.5 ml of LS buffer (20 mM Tris pH 7.4, 200 mM LiCl, 1 mM EDTA) and incubated at room temperature for 5 min without rotation. Using a magnetic rack, LS buffer was removed and beads centrifuged at 3000 rpm for 10 s and residual LS buffer was removed. Beads were re-suspended in 60 μl of elution buffer (20 mM Tris pH 7.4, 1 mM EDTA) per tube and incubated at 92°C for 4 min, with shaking at 450 rpm. Tubes were placed in a magnetic rack and supernatants pooled into fresh 2 ml tubes. The elution step was repeated and eluates were pooled with first elution for each condition and RNA concentration measured using a NanoDrop. RNA was then sodium acetate/ethanol precipitated. Pellets were resuspended in 45–55 μl elution buffer plus 1 mM MgCl_2_, 125 U Benzonase (Sigma-Aldrich catalogue # E104-25KU) and 300 U RNase I (Ambion catalogue # AM2295). Samples were incubated at 37°C for 2 h to digest the RNA. 5× SDS PAGE loading buffer was added to the samples.

### Sample preparation and mass spectrometry

Samples were subjected to SDS-PAGE and gels were stained in ProtoBlue™ Safe Colloidal Coomassie Blue G-250 stain (National Diagnostics) as per manufacturer's instructions. Gels were then prepared for mass spectrometry analysis as follows: lanes were equally sectioned and gel slices were alternately washed in 50 mM NH_4_HCO_3_ and 100% (v/v) acetonitrile three times. Samples were reduced in 10 mM dithiothreitol (for 20 min, 56°C) and alkylated in 100 mM iodoacetamide, prior to rinsing in 50 mM NH_4_HCO_3_/100% (v/v) acetonitrile. Gel pieces were soaked in trypsin digestion buffer (25 mM NH_4_HCO_3_ containing 11.11 μg/ml of Modified Trypsin (Promega Corporation, USA)). Digests were incubated overnight at 30°C. Trifluoroacetic acid was added to each digest at 0.2% (v/v) final concentration and reactions incubated for 1 h at room temperature to extract protein. Samples then lysophilized in a Savant DNA Speed Vac (Thermo Scientific, USA). Lysophilized peptides then resuspended in 5% (v/v) formic acid/acetonitrile at a ratio of 9:1. Samples were spiked with two standards, yeast ADH1 (P00330) and bovine serum albumin (P02769) (MassPREP standards, Waters Corporation, UK) to a final concentration of 20 fmol/μl. Peptides were then separated by nanoflow liquid chromatotgraphy coupled to a Synapt G2S mass spectrometer for analysis (NanoAcquity UPLC system and Synapt G2S mass spectrometer, Waters Corporation, UK), using a 25 cm by 75 μm I.D., 1.7 μm BEH130 C18 column. 2 μl sample injections were separated using a 90 min reversed phase, 3–40% (v/v) acetonitrile gradient, run at 0.3 μl/min. Mass spectrometry analysis was performed in a data-independent manner, using ion mobility HDMSE, with IMS wave velocity in the helium cell set to 650 m/s. The mass spectrometer stepped between 4 eV (low energy) and 20–50 eV (elevated collision energy) in the gas cell, with a scan time of 1 second and a mass range of 50–2000 *m*/*z*. Protein identifications and absolute quantification information were extracted from the raw data files using ProteinLynx Global Server (PLGS version 3, Waters Corporation, UK) in combination with ISOQuant (Kuharev and Tenzer, Germany, open source under http://www.immunologie.uni-mainz.de/isoquant/). Data was then processed with energy thresholds set to 135 and 30. The human UniProt database, including reverse sequences (UniProtKB/SwissProt, release 2014_05, 11.06.2014, 20265 entries) was used in PLGS, with peptide mass tolerance and fragment mass tolerance set to automatic, with an allowed maximum of one missed cleavage. Ion matching requirements set to one or more fragments per peptide, three plus fragments per protein and one or more peptides per protein, with a False Discovery Rate (FDR) of 1%. Processed, database searched PLGS data files were loaded into ISOQuant for quantitative analysis using an FDR of 0.1%, only returning an absolute fmol amount of protein if three reliable peptide hits were available for quantification (TOP3 method). This software used first past matches, thereby ignoring any peptides generated by in-source fragmentation or modified peptides. ADH1 internal standards were used to calculate absolute amounts of identified proteins. Software did not identify splicing isoforms of proteins independently of one another, instead identifying them as a single protein based on peptides. Data from TOP3 ISOQuant analysis was exported to excel for further fold-change and statistical analysis. Non-human spike-ins and proteins with missing observations were removed. The resulting list of 214 proteins were adjusted for batch effects using the SVA package (60) in R. Statistical analysis was then performed using the openly available limma Bioconductor package (61). Method adapted from (62).

### Endogenous immunoprecipitation of LARP1 or PABPC1

HeLa cells were seeded at 1.8 × 10^6^ per plate. After 24 h, the media was supplemented with 2% (v/v) foetal bovine serum, 2mM l-glutamine for 30 min followed by 1 h with DMSO or 200 nM Torin1. Meanwhile, protein A/G beads were pre-coupled to antibodies as follows; using 100 μl bead slurry (Protein A/G plus agarose SC-2003) per IP: bead slurry was washed twice with 1 ml IP lysis buffer (20 mM Tris–HCl pH 7.5, 150 mM NaCl, 15 mM MgCl_2_, 0.5% (v/v) nonidet P40/Igepal, 1 mM EDTA, 0.5% (w/v) sodium deoxycholate, 0.1% (v/v) 2-mercaptoethanol, protease inhibitor cocktail tablet (Roche Applied Sciences catalogue # 11836170001), 1 mM β-glycerophosphate, 1 mM sodium fluoride and RNasin Plus RNAse inhibitor (40 U/ml)) and resuspended to initial volume. Beads were pre-coupled in IP lysis buffer with 3 μg per IP of either Rabbit IgG (0.4 μg/μl), PABPC1 antibody (1 μg/μl) or LARP1 Antibody (0.2 μg/μl) for 2 h at 4°C with end-over-end rotation. Pre-incubated beads were spiked with 1:100 (v/v) yeast tRNA for 30 min at 4°C. Beads then washed three times with 1 ml of lysis buffer and 100 μl beads used per IP. Following treatments, cells were washed twice with 8 ml volume of PBS then lysed in 2 ml of IP lysis buffer per plate. Where RNase I was used (Supplementary Figure S9), 150 U/ml was added to the lysis buffer prior to lysis (and RNasin Plus RNase inhibitor was not added). Lysates were passed through a 21G needle three times before equilibration by end-over-end rotation for 5 min at 4°C. Lysates were then spun down for 15 min at 4°C at 13 000 rpm, supernatants collected and pooled. Protein concentration determined by Bradford and lysates normalized for protein concentration. 100 μl of lysate reserved for western and 100 μl for input RNA (added to 1 ml TRIzol Reagent (from Thermo Fisher Scientific catalogue # 15596018)). Beads were incubated with 1.5 ml normalized protein extract at 4°C with end-over-end rotation for 90 min. Beads washed four times for 5 min with end-over-end rotation with 1 ml of lysis buffer. At the last 1 ml wash: beads were pooled for each treatment and split for RNA and protein analysis: 200 μl for protein analysis- centrifuged briefly, supernatant removed and resuspend in 60 μl 1× sample buffer; 800 μl for RNA analysis- wash buffer was removed and 1 ml of Trizol was added. RNA extraction was then performed as described below.

### RNA extraction

RNA extraction was carried out using Trizol reagent (from Thermo Fisher Scientific catalogue # 15596018) following the manufacturer's instructions. Glycogen was used as a carrier to aid RNA precipitation. Trizol extraction was followed by phenol: chloroform extraction and overnight sodium acetate/ethanol precipitation at –20°C.

### Endogenous immunoprecipitation of LARP1 with UV254 cross-linking

HeLa cells were seeded and treated as for endogenous IPs. Following treatments cells were washed twice with 8 ml volume of PBS. Plates (with no lid or PBS) were placed on a tray of ice in 254 nm UV oven and irradiated at 150 mJ/cm^2^ (two plates at a time). 2 ml of IP lysis buffer was added per plate and lysates were collected as for endogenous immunopreciptations above. Protein concentration was determined by Bradford Assay and lysates normalized for protein concentration. 100 μl of lysate was reserved for western blotting and 100 μl for input RNA (treated as mentioned later). Protein A/G beads precoupled to antibodies were spun down and resuspended in 100 μl lysis buffer per treatment. 100 μl aliquots were made for IPs and 12 μl tRNA was spiked into each tube. Lysates were incubated with indicated antisera coupled beads at 4°C on a wheel for 90 min. Beads were washed twice for 5 min with end-end rotation with 1 ml high salt buffer (20 mM Tris–HCl pH 7.5, 350 mM NaCl, 15 mM MgCl_2_, 0.5% (v/v) IGEPAL, 1 mM EDTA, 0.5% (w/v) sodium deoxycholate, 0.1% (w/v) SDS). Beads were then washed twice for 5 min with end-end rotation with 1 ml iso buffer (20 mM Tris–HCl pH 7.5, 150 mM NaCl, 15 mM MgCl_2_, 1 mM EDTA and 7 mM β-mercaptoethanol). At last, 1 ml wash beads were pooled for each treatment and split for RNA and protein analyses: 700 μl for RNA and 240 μl for protein. Supernatants were removed and beads for RNA analysis resuspended in 100 μl proteinase K solution (iso buffer containing 80 U/ml RNasin Plus RNAse inhibitor, 1% (w/v) SDS and 200 μg/ml proteinase K (Invitrogen catalogue # 25530049). 100 μl of each input were also proteinase K treated by adding 200 μg/ml proteinase K solution. Samples were incubated at 37°C for 40 min with shaking at 800 rpm then added to 1 ml Trizol LS and frozen at –80 prior to RNA extraction. Beads for protein analysis were re-suspended in 55 μl iso buffer. Premixed RNAse/DNAse (50 U Benzonase, 50 U RNase I and 50U RNAse A) was added and incubated at 37°C for 2 h to digest RNA. One hundred microlitres of each input was also incubated with 50 U of the RNAses. 20 μl 5× SDS PAGE loading buffer was added to IPs and 40 μl to inputs for western blot analysis.

### Transient transfection and flag pulldowns

HeLa cells were seeded at 1.6 × 10^6^ per plate. After 24 h, cells were transfected with 5 μg of vector (as indicated in figure legends). Twenty four hours following transfection cells were Torin1/DMSO treated, harvested, lysed and cleared supernatants prepared and normalized for protein concentration as for endogenous IPs (above). Normalized lysates were incubated with end-end rotation with 50 μl Anti-FLAG^®^ M2 magnetic beads (Sigma-Aldrich catalogue # M8823) for 90 min at 4°C. Beads were then washed 4 times for 5 min in IP lysis buffer (buffer as described for endogenous IPs). At the last 1 ml wash beads were split for RNA and protein analysis as described in the endogenous IP protocol above. For competitive elution, performed for Flag RNA binding protein experiments shown in Supplementary Figure S2: Immunorecipitations were performed as above, with the exception that following the fourth wash, beads were resuspended in 100 ul of Flag elution buffer (IP lysis buffer containing 200 ng/ul 3× FLAG^®^ peptide (Sigma-Aldrich catalogue # F4799) and elution performed as manufacturers protocol. Eluates were split for RNA and protein analysis as described previously in the endogenous IP protocol.

### mRNA arrays and analysis

Resuspended RNA from endogenous pulldowns was labelled and hybridized to 60K human gene expression whole genome microarrays (Agilent Technologies, Berkshire, UK), following the manufacturer's protocol and as described (63). 100 ng input RNA, and the contents of 10 μl IP RNA (from a total of 25 μl) was Cy-3 labelled using Agilent Low Input Quick Amp 1-colour Labelling Kit (Agilent). The level of dye incorporation was measured using a Nanodrop ND1000 spectrophotometer (LabTech, Sussex, UK). 600 ng Cy-3 labelled input sample, 600 ng Cy-3 labelled IP mRNA (antibody-treated samples) and the equivalent volumes of vehicle control samples were taken and all fragmented using fragmentation buffer from the Agilent Gene Expression Hybridization Kit (Agilent) for 30 min at 60°C. Fragmented samples were mixed 1:1 with Hybridization Buffer (v/v) and hybridized to a 60K high-density oligonucleotide microarray overnight (G4858-039494 (Agilent SurePrint G3 Human GE v2 8 × 60K Microarray 039381 (Feature Number version)). Microarrays were loaded as per the manufacturer's instructions and hybridization was performed at 65°C, in an Agilent Hybridization Oven with rotation set to 10 RPM. Following hybridization, the microarray slides were washed in Gene Expression wash buffers 1 and 2 (Agilent) and immediately scanned using a DNA Microarray Scanner (Model G2505C, Agilent Technologies). Array analysis performed using limma, using normalized expression background correction, quantile normalization, filtered for spots with intensity >1.5 of negative control probes, FDR <0.05 applied to linear model fitting. Enrichment in the IPs was calculated as fold change of signal in the immunoprecipitated sample versus the corresponding input RNA samples. Those mRNAs considered to be enriched in the IP had a log FC > 0.5 and FDR < 0.05. RNAs binding to LARP1 or PABPC1 were divided into the following groups for subsequent analysis: ‘induced bound’: log FC ≤ 0 in control and log FC > 0.5 in Torin1-treated; ‘constitutively bound’: log FC > 0.5 in both control and Torin1-treated, ‘unbound’: log FC < 0 FDR < 0.05 in both control and Torin1 conditions. For Figures 2E and 5F and Supplementary Figure S5, to provide comparable groups to constitutively bound and induced bound mRNAs, unbound mRNAs were further broken down into ‘constitutively depleted’: log FC < –0.5 in control and Torin1-treated and ‘induced depleted’: FDR>0.05 in control and log FC < –0.5 in Torin1-treated.

### Polysome sucrose density gradient centrifugation and fractionation for RNA analysis

HeLa cells were cultured and treated as previously and incubated with 0.1 mg/ml cycloheximide at 37°C 3 min prior to lysis, rinsed twice in PBS containing 0.1 mg/ml cycloheximide and scraped into chilled lysis buffer (15 mM Tris–HCl pH 7.5, 0.3 M NaCl, 15 mM MgCl_2_, 0.1 mg/ml cycloheximide, 1 mg/ml heparin and 1% Triton-X-100). Cell lysates were then placed on ice for 10 min to allow lysis to occur. Nuclei and cell debris were removed by centrifugation at 4°C for 5 min at 13 000 rpm. Supernatants were then loaded onto 10–50% sucrose gradients (containing 15 mM Tris–HCl (pH 7.4), 15 mM MgCl_2_, 0.15M NaCl, 0.1 mg/ml cycloheximide, and 1 mg/ml heparin) in Sorvall PA 12 ml tubes. Gradients sedimentation was performed at 38 000 rpm for 2 h in an SW40Ti at 4°C. Sucrose gradients were fractionated at 1 ml/min on an ISCO fraction collector apparatus. 1 ml fractions were collected directly into 3 ml 7.7 M guandine–HCl and the RNA precipitated by addition of 4 ml volume of 100% ethanol. The RNA was resuspended in water and further purified by 2.5 M LiCl precipitation followed by sodium acetate/ethanol precipitation.

### Polysome sucrose density gradient centrifugation and fractionation for analysis of protein distribution

HeLa cells were transiently transfected with 5 μg per plate Flag tagged LARP1 2-1019 wild type (WT), PAM2 L423A/F428A double point mutant (PAM2M), or bacterial alkaline phosphatase (BAP) flag tagged control constructs. Twenty-four hours following transfection, medium was supplemented with 2% serum, 2 mM l-Glut for 30 min followed by 1 h with DMSO (Control) or 200 nM Torin1. Cells were treated with 100 μg/ml cycloheximide for the last 3 min prior to lysis. After PBS/cycloheximide washes cells were scraped into chilled hypotonic lysis buffer (5 mM Tris–HCl pH 7.5, 1.5 mM KCl, 2.5 mM MgCl_2_, 0.1 mg/ml cycloheximide, 0.5% (v/v) Triton-X-100, 0.5% (w/v) sodium deoxycholate with 0.1% (v/v) 2-mercaptoethanol, protease inhibitors (Roche Applied Sciences catalogue # 11836170001) and 40 U/ml RNasin Plus RNase). Nuclei and cell debris were removed by centrifugation at 4°C for 5 min at 13 000 rpm. Supernatants were then loaded onto 10–50% sucrose gradients made up in buffer containing 20 mM HEPES–KOH pH 7.5, 100 mM KCl, 5 mM MgCl_2_, 100 μg/ml cycloheximide. Gradient sedimentation and fractionation were performed as with RNA polysome gradient method. Western blotting was performed on each fraction.

### Ribosome profiling analysis

Translational efficiency (TE) data from (43) was sorted into LARP1 RIP mRNA groupings: constitutively bound (larp_constitutive), mTOR inhibition induced bound (larp_induced) and unbound mRNAs, for each of four conditions: wild type (WT) or LARP1 knockout (KO) HEK293T cells with either control (DMSO) or Torin1 treatment. This analysis was also performed for LARP1–PABPC1 bound mRNAs. Ribosome occupancy for LARP1 and PABPC1 bound mRNA groupings was calculated from ribosome profiling data for three replicates in HEK293 cells in control conditions, obtained from Wilczynska and Gillen (64). Figures 3D and 5F show normalized ribosome occupancy. This is the ribosome protected fragments (RPFs) at each codon position normalized for mRNA abundance for indicated mRNA groups. To get a global view across the whole CDS, the RPF coverage across each transcript CDS has been length normalized.

### RT-qPCR

RNA was reverse transcribed by using SuperScript III (Invitrogen) and Random primers (Invitrogen). Real-time PCR was carried out by using the Applied Biosystems 7500 Fast Real-Time PCR System and Fast SYBR™ Green Master Mix (Applied Biosystems) using the manufacturer's protocol. Gene specific primers used are shown in Supplementary Table S4.

### SDS-PAGE and western blotting

Protein samples were subjected to SDS-PAGE using the Bio-Rad protein mini gel system using resolving gels of 10%, 12% or 15% (w/v) acrylamide depending on the sizes of the proteins being blotted. Proteins were transferred from gels onto 0.4 μm nitrocellulose membrane using the Bio-Rad protean II wet transfer system at 100 V for 2 h. Membranes were then blocked in TBS-Tween 5% (w/v) milk for 30 min to 1 h. Primary antibodies were used at the recommended dilutions in TBS–Tween 5% milk (w/v) for total antibodies and TBS–Tween 5% BSA (w/v) for phospho-antibodies. IRDye^®^ conjugated secondary antibodies (LI-COR) were used at a dilution of 1:10 000 and western blots were imaged using the LI-COR Odyssey imaging system and were analysed using Image Studio software Version 2.1.

### Silver staining of SDS-PAGE gels

SDS-PAGE gels were rinsed in water and then fixed for 2 h (50% (v/v) EtOH, 12% (v/v) Acetic Acid, 0.05% (v/v) formaldehyde), washed for 3 × 15 min in 50% (v/v) EtOH, put into sensitization solution for 1 min (0.02% (v/v) Na_2_S_2_O_3_), washed twice in water followed by 20 min in staining solution (0.2% (w/v) AgNO_3_, 0.075% (v/v) formaldehyde), washed twice in water for 30 s followed by 5–15 min in development solution (3% (v/v) Na_2_CO_3_, 4 mg/l Na_2_S_2_O_3_, 0.05% formaldehyde). Developing was halted using 1% (w/v) glycine for 30 min followed by washing with water. Gels were imaged using a LI-COR Odyssey imager and Image Studio software.

### Coomassie staining of SDS-PAGE gels

SDS-PAGE gels were placed into 10mL of InstantBlue™ (Sigma ISB1L) for 1–16 h with gentle rocking. Gels were then rinsed for 4 × 15 min before imaging on the LI-COR Odyssey system. Gels used for mass spectrometry were stained as detailed in the sample preparation and mass spectrometry methods section.

### m^7^GTP pulldowns

50 μl of m^7^GTP-agarose (Jena Bioscience catalogue # AC-155L) per pulldown was gently centrifuged, storage buffer aspirated and beads resuspended in an equal volume of m^7^GTP lysis buffer (50 mM MOPS/KOH pH7.2, 50 mM NaCl, 2 mM EGTA, 5 mM EDTA, 7 mM β-mercaptoethanol, 50 mM β-glycerophosphate, 50 mM NaF, 1% (v/v) IGEPAL, 1% (v/v) Triton-X-100, protease inhibitor tablet (Roche Applied Sciences catalogue # 11836170001)). 600 μl of each normalized lysate was incubated with 50 μl of m^7^GTP-agarose (pre-equilibrated in lysis buffer). Pulldowns were performed for 1 h at 4°C with end over end rotation. Beads were gently pelleted for 20 s at 4000 rpm, the supernatant removed and 1 ml wash buffer added ((50 mM MOPS/KOH pH 7.2, 50 mM NaCl, 2 mM EGTA, 5 mM EDTA, 7 mM β-mercaptoethanol, 50 mM β-glycerophosphate, 50 mM NaF). The wash step was repeated twice and beads were resuspended in 70 μl sample buffer and heated at 95°C for 5 min prior to running on SDS-PAGE gels and western blotting.

### Northern blotting

DNA probes for northern blotting were generated from HeLa cDNA by Taq polymerase PCR using the primers listed in Supplementary Table S4 (where (N) designates northern probe primers). DNA was gel extracted using the QIAquick gel extraction kit (Qiagen) as per the manufacturer's protocol. For northern blotting RNA was subjected to size separation on an RNAse free 1% agarose formaldehyde–MOPS gel alongside 2 and 10 kb RNA markers. RNA was passively transferred to zeta probe membrane (Bio-Rad) in 20x SSC buffer (3 M NaCl, 0.3 M sodium citrate solution). RNA was cross-linked to the membrane using 254 nm Stratalinker set on 1200. The membrane was stained in methylene blue solution (0.02% (w/v) methylene blue and 0.3 M Na acetate (pH 5.2)) and then washed several times in water, followed by several washes in 1× SSC containing 1% SDS and two rinses in water. Northern blots were pre-hybridized in 5 ml Church–Gilbert's solution at 65°C for 30 min in a hybridization oven. Meanwhile, 50 ng of DNA probes were then radiolabelled with αP32 dCTP using Klenow enzyme (New England Biolabs). Probes were then passed through G-50 columns (GE Healthcare) to remove unincorporated nucleotides. Filtered probe was denatured, cooled and 25 μl of end-labelled probe added to Church-Gilberts Solution (140 mM Na_2_PO_4_, 70 mM NaH_2_PO_4_, 7% SDS) and each blot incubated in a hybridization oven overnight at 65°C. Blots were then washed by rotation for 15 min at RT twice in each of three wash solutions: Wash solution 1 (2× SSC, 0.1% SDS); Wash solution 2 (0.5× SSC, 0.1% SDS); Wash solution 3 (0.1× SSC, 0.1% SDS). Blots were then wrapped in cling film and placed in a developing cassette with a blank imaging plate overnight. Blots were visualized on a phosphorimager.

### Cloning and DNA overexpression constructs

cDNA was made from HeLa cells by Trizol extraction of RNA followed by reverse transcription. Generated cDNA was then used as a template to amplify SERBP1, TRIM25 and PWP2 using Phusion high fidelity DNA polymerase (New England Biolabs) and primers listed in Supplementary Table S5. SERBP1 and PWP2 were cloned into LEICS-12 N-HIS10/3× Flag vector and TRIM25 was cloned into LEICS-49 C-HIS4/3× Flag by the University of Leicester protein expression laboratory. LARP1 was sub-cloned from pCMV5-LARP1 (NM015315.3) into p3xFlag-CMV™-7.1 expression vector (Sigma-aldrich E4026) (restriction digested using Hind3 and XBA1 followed by gel extraction and ligation, to produce n-terminal Flag-tagged LARP1 purposefully omitting the initiating methionine. The primers used are listed in Supplementary Table S5. p3xFlag-CMV™-7-BAP control expression vector was purchased from Sigma (C7472). For generation of pET-SUMO PABPC1, cDNAs coding for PABPC1 (1–636) were generated using standard PCR using PABPC1 primers listed in Supplementary Table S5. PCR product was subsequently cloned into pET-SUMO vector using the BsaI and NotI restriction sites. All CDS open reading frames were sequenced fully for each construct.

### Site directed mutagenesis

Site directed mutagenesis was performed using Quickchange lightning (Agilent) according to the manufacturer's protocol. Primers used for mutagenesis are shown in Supplementary Table S5. PCRs were Dpn1 treated and used to transform *E. coli* DH5α. For the LARP1 R840E/Y883A double point mutant, mutagenesis was performed sequentially post sequencing of the correct clone.

### Generation of RPS16 promoter and 5′UTR destabilized firefly luciferase constructs

Initial RPS16 TOP reporter containing the promoter element and whole 5′UTR fused to firefly luciferase pGL3-enhancer vector backbone was a kind gift from Anders Lund (initial construct described (65)). The region containing these elements was amplified and inserted into a pGL3 vector modified in the 3′UTR to contain 14× MS2-repeats between Bsa1/BsrG1 sites. The parental vector was constructed using MS2 sites that were PCR amplified from pSL1180-MS2-12X (66) (a kind gift from Prof. Robert H. Singer, Albert Einstein College of medicine, Yeshiva University.) and inserted between two existing MS2 sites using EcoR1/NHE1 restriction sites as described (67). To generate a faster turnover luciferase protein, destabilization elements hCL1 and hPEST were cloned into the c-terminus of the ORF from the pGL4.12 vector (Promega E6671) by firstly performing PCR using pfu turbo (Agilent) and the following primers with the pGL4.12 template:

hCL1 for - CAAGAAGGGCGGCAAGATCGCCGTGAATTCTGCTTGCAAGAACTGGTTCAG

PEST rev - GGATCCCCCCTAGAGTATTACTCTAGAATTAGACGTTGATCCTGGCGCTGGCG

The 233 bp product was then gel extracted and used as a megaprimer in a second PCR reaction using the Quickchange lightning kit (Agilent 210519) and the RPS16 TOP reporter as template. Reactions were then Dpn1 treated and transformed into *E. coli*. Miniprep DNA extracted and sequenced. The resulting plasmid is known as Pgl3 RPS16 WT TOP hCL1 PEST. RPS16 MUT TOP hCL1 PEST was generated by site directed mutagenesis using the RPS16 MUT for and rev primers in Supplementary Table S5 to generate a mutant that no longer contained the TOP motif and C-cap: **C**CUUUUCC but instead **G**AGUGACC.

### Recombinant protein production

Recombinant human full length LARP1 (1–1096) was expressed and purified from *E. coli* at Viva Biotech using the following protocols. His 6-TEV-LARP1 (1–1096) was codon optimized and cloned into pET21a (Novagen) via the NdeI/XhoI restriction sites. pET21a-His 6 -TEV-LARP1 (1–1096) was transformed into BL21 (DE3) GOLD and grown on LB agar with 100 μg/ml ampicillin and incubated at 37°C overnight. A colony was used to inoculate a starter culture in LB media (supplemented with 100 μg/ml ampicillin) and incubated overnight at 37°C with shaking (220 rpm). Cultures were expanded in Terrific Broth (500 ml), supplemented with 100 μg/ml ampicillin, and were incubated at 37°C with shaking (220 rpm). When cultures reached an OD_600_ of 0.6, the temperature was reduced to 18°C and protein induced for 16 h with 0.5 mM IPTG. Cells were harvested by centrifugation at 4000 g for 20 min. Cell pellets were flash frozen in liquid nitrogen and stored at –80°C. All purification steps were performed on ice or at 4°C. Cell pellets (typically ∼ 100 g) were resuspended at 10% (w/v) in Lysis Buffer (50 mM Tris–HCl pH 7.5, 500 mM NaCl, 20 mM imidazole, 10% (v/v) glycerol, Complete Ultra EDTA-free protease inhibitor tablets (used as manufacturer's instructions – Roche Applied Sciences catalogue # 11836170001)) and Benzonase ((Sigma-Aldrich catalogue # E104-25KU) 1 μl per 10 ml of Lysis Buffer). Lysates were sonicated and clarified by centrifugation at 47 000 g for 1 h followed by filtration through a 0.2 μm filter. The supernatant was loaded onto 2 × 5 ml HisTrap HP columns, pre-equilibrated with Loading Buffer (50 mM Tris–HCl pH 7.5, 500 mM NaCl, 10% glycerol, 20 mM imidazole). The column was washed with 10 column volumes (CV) of Loading Buffer followed by 25 CV of High Salt Buffer (50 mM Tris–HCl pH 7.5, 1 M NaCl, 10% glycerol, 20 mM imidazole). The column was finally washed with 5 CV of Low Salt Buffer (50 mM Tris–HCl pH 7.5, 150 mM NaCl, 10% (v/v) glycerol, 20 mM imidazole). His-TEV-LARP1 was eluted from the column using 5 CV of 50 mM Tris–HCl pH 7.5, 150 mM NaCl, 10% glycerol, 300 mM imidazole. Fractions containing His-TEV-LARP1 were pooled and diluted 1 in 5 with Heparin A Buffer (50 mM Tris–HCl pH 7.5, 10% glycerol, 1 mM DTT). Diluted protein sample was loaded onto 2 × 5 mL HiTrap Heparin HP columns, pre-equilibrated with Heparin Running Buffer (50 mM Tris–HCl pH 7.5, 40 mM NaCl, 10% glycerol, 1 mM DTT). The column was washed with 30 CV of this buffer followed by elution using a linear salt gradient of 40 mM to 1 M NaCl in Heparin Running Buffer over 25 CV. Fractions containing His-TEV-LARP1 were pooled and concentrated to approximately 1–2 ml using a Millipore 30 kDa MWCO centrifugal concentrator. This sample was loaded onto a HiLoad 16/600 Superdex 200 column, pre-equilibrated with SEC Buffer (50 mM Tris–HCl pH 7.5, 500 mM NaCl, 10% glycerol, 1 mM DTT). Following identification by SDS-PAGE analysis, the His-TEV-LARP1 containing fractions were pooled, flash frozen in liquid nitrogen and stored at –80°C.

Recombinant human PABPC1 (1–636) was expressed and purified from *E. coli* using an N terminal His-SUMO tag. His-SUMO-PABPC1 (1–636) was transformed into Rosetta2 (DE3) and grown on LB agar, supplemented with 50 μg/ml Kanamycin and 33 μg/ml Chloramphenicol, and incubated at 37°C overnight. Cell culture and induction were carried out using identical protocols to His-TEV-LARP1. Purification of PABPC1 was carried out on ice or at 4°C unless stated. Cell pellets were resuspended in 10% (w/v) in Lysis Buffer (as used for LARP1 with the exception of a higher, 40 mM imidazole concentration). Lysis by sonication was carried out and cell lysates centrifuged at 47 000g for 1 h, followed by filtration through a 0.2 μm filter. Supernatant was loaded onto a 5 ml HisTrap HP column, pre-equilibrated with Loading Buffer (50 mM Tris–HCl pH 7.5, 500 mM NaCl, 10% (v/v) glycerol, 40 mM imidazole) and washed first with 10 CV of Loading Buffer and then with 25 CV of High Salt Buffer (50 mM Tris–HCl pH 7.5, 1 M NaCl, 10% (v/v) glycerol). His-SUMO-PABPC1 was eluted from the column with 50 mM Tris–HCl pH 7.5, 150 mM NaCl, 10% (v/v) glycerol, 250 mM imidazole. The eluted peak was pooled and incubated overnight with SUMO protease (Life sensors) at 4°C using 10 U/1 mg of target protein. The resulting sample was diluted 1 in 6 with Heparin A Buffer (50 mM Tris–HCl pH 7.5, 10% (v/v) glycerol, 1 mM DTT). Sample was loaded onto a 5 ml HiTrap Heparin HP column, pre-equilibrated with Heparin Running buffer (50 mM Tris–HCl pH 7.5, 30 mM NaCl, 10% (v/v) glycerol, 1 mM DTT) and washed with 30 CV of the same buffer. PABPC1 was eluted in a linear 30 mM to 1 M NaCl gradient in Heparin Running Buffer over 20 CV. Fractions containing PABPC1 were pooled and concentrated to ∼1–2 ml using a Millipore 30kDa MWCO centrifugal concentrator. This sample was loaded onto a HiLoad 16/600 Superdex 200 column, pre-equilibrated with SEC Buffer (50 mM Tris–HCl pH 7.5, 500 mM NaCl, 10% (v/v) glycerol, 1 mM DTT). Following identification by SDS-PAGE analysis, PABPC1-containing fractions were pooled and passed through a 5 ml HisTrap HP column equilibrated in SEC Buffer to remove residual uncut SUMO-tagged protein. Recovered tag-free protein was pooled, aliquoted, snap frozen and stored at –80°C.

### Immunoprecipitations with recombinant proteins

To study the PABPC1-LARP1 interaction *in vitro*, 2.5 μM of recombinant His-LARP1 and PABPC1 were incubated ±2 μM Dye680-labelled A20-RNA (IBA life science) for 1.5 h at room temperature in 50 mM Tris–HCl, pH 7.5, 500 mM NaCl, 10% (v/v) glycerol. Per reaction 50 μl Dynabeads Protein G (Thermo Fisher Scientific) were washed three times with NP-buffer (20 mM HEPES, 100 mM KCl, 0.2 mM EDTA, 1 mM TCEP, 0.1% (v/v) IGEPAL (Sigma-Aldrich)) and coated with 5 μg of 6× Histag (abcam ab18184) or IgG-control (sc-137148) antibody for 30 min in NP-buffer at room temperature. Coated beads were washed three times in NP-buffer and incubated with protein samples for 15 min at room temperature. Beads were then washed three times with NP-buffer, mixed with SDS gel loading buffer, incubated at 95°C for 3 min and applied to denaturing polyacrylamide gel electrophoresis. After electrophoresis, gels were stained with Coomassie and bands visualized using a Licor Odyssey scanner.

### RNase degradation assay

To investigate the activity of RNase I, 1 μM Dye680-labelled A20 RNA (IBA life science) was incubated with 0.25 mg/ml RNase I in the presence of 8 μM competitor RNA (5′-GAAAAAAUUAAAAAAUUAAAAAAC-3′, IDT) at room temperature in lysis buffer (20 mM Tris–HCl, pH 7.5, 150 mM NaCl, 15 mM MgCl_2_, 0.5% (v/v) IGEPAL, 1 mM EDTA, 1 mM NaF, 1 mM glycerophosphate, 1× protease inhibitors (Roche, EDTA-free)). Before the addition of RNase I and after 1 and 2.5 h reaction time aliquots were taken and mixed 1:1 with stop solution (0.5× TBE, 10 mM EDTA, 0.1% (w/v) SDS, 85% (v/v) formamide). Samples were heated for 2 min at 95°C and loaded on acryladmide–8M–urea TBE gels. After electrophoresis bands were visualized using a Licor Odyssey scanner. A dye680-labelled A2 dinucleotide was used as a size marker.

### siRNA transfections and RPS16 5′UTR TOP reporter luciferase assays

SiRNA transfections were performed using Lipofectamine^®^ RNAiMAX transfection reagent (Thermofisher Scientific 13778150) diluted in OptiMEM (GIBCO Cat # 11058) at stated final concentrations. Negative control siRNA was ON-TARGETplus non-targeting siRNA #3 (Thermo Scientific, catalogue # D-001810-30). siRNA against *LARP1* (NM_033551.3) was custom ordered from Invitrogen (Fwd 5′gugaacccguggacuaagaac 3′ Rev 5′ guucuuaguccacggguucac 3′). HeLa cells were seeded at a density of 50 000 cells/well on top of the reverse-siRNA mix. Per well the transfection mix was as follows: 0.6 μl 20 nM siRNA plus 3 μl RNAiMAX prediluted in 46.4 μl OptiMEM media. Transfection mix (50 μl) was pipetted into each well prior to 550 μl of cells, to give a final concentration of 20 nM siRNA. Replicate plates were plated to allow for luciferase assays and RNA extractions from set 1 and western blots from set 2. Medium was changed after 24 h and after 48 h cells were transfected with reporter plasmids using GeneJammer reagent as follows: Per well, 1.8 μl of GeneJammer reagent was diluted with 18.2 μl OptiMEM and incubated at room temperature for 5 min. Then 500 ng per well of RPS16-PEST-CL reporter and 100 ng per well of control pRL-SV40 vector (Promega catalogue # E2231) used as control vector were added and transfection mix incubated for 30 min at RT. Then 20 μl of transfection mix was added to the relevant wells and left for 24 h. Medium was replaced with 500 μl of medium containing 200 nM Torin1 or DMSO (control) and FBS/l-glutamine (as previously described) for 2h. Medium was aspirated and wells rinsed once with 2 mL PBS. PBS was then aspirated and 100 μl of passive lysis buffer added per well for luciferase plates and 75 μl IP lysis buffer for western wells. Plates were incubated for 15 min with gentle rocking and then frozen at –80°C. For luciferase assays plates were defrosted and 10 μl of passive lysis buffer lysate were assayed in duplicate for each of three triplicate wells using the Dual-Luciferase Reporter Assay System (Promega) on a GloMax 96 Microplate Luminometer (Promega) using Promega protocol with 50 μl injections of each reagent. Relative luciferase activity was calculated as a ratio of firefly luciferase to Renilla luciferase (with normalization to an internal control to account for inter-experimental variance). 40 μl of the same lysate was used for RNA extractions and RT-qPCR analysis was performed using luciferase primers listed in Supplementary Table S4. For corresponding westerns, plates were defrosted and 3 × 75 μl lysates (triplicate wells) were pooled and spun at 13 000 rpm 20 min and lysates normalized for protein concentration following Bradford assays. Western blots were performed using indicated antibodies.

### Polyadenylation test (PAT)

Polyadenylation assays were performed and analysed on mRNA purified from HeLa cell lysates treated with/without mTOR inhibition as described in (68). Primers used are listed in Supplementary Table S6.

### Statistical analysis

Unless otherwise stated, error bars represent mean ± 1 SD of the replicates, with numbers of independent experiments (*N*) indicated in the figure legends. Statistical testing was performed using the Student's *t*-test, unpaired, two-tailed. In all cases significance is indicated as follows: *P ≤ 0.05, **P ≤ 0.01, ***P ≤ 0.001.

## RESULTS

### Identification of mTOR-regulated RNA-binding proteins

**Figure 1.**
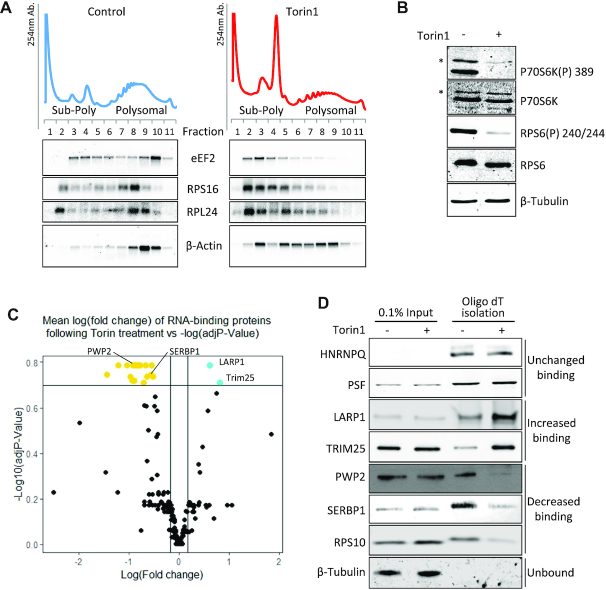
Changes in RNA-binding proteins upon mTOR inactivation. (A–D) HeLa cells were treated with 200 nM Torin1 or DMSO as control. (**A**) Polysome profiling was performed and RNA from fractions was analysed by Northern blot. Representative UV traces from one of two independent experiments and corresponding blots from gradient fractions using probes against indicated RNAs are shown. B–D: Whole cell RNA-binding protein (RBP) capture using oligo(dT) affinity isolation. (**B**) Representative western blots of lysates from one of three experiments used for oligo(dT) pulldowns in C and D to show mTOR inactivation and equal loading using indicated antisera. Asterisks denote p85S6K. (**C**) Plot of mean fold change of proteins identified in control (DMSO carrier) versus Torin1 (mTOR inhibited) RBP capture (using TOP3 unique peptides for quantitation). Cut-offs shown on graph are log FC > 1.3, adjusted *P*-values <0.2 using Benjamini–Hochberg adjusted P-value. Following Torin1 treatment: gold = RBP decreasing binding; cyan = RBP increasing binding; black = no significant change in binding. Three independent experiments performed. (**D**) Validation of changes in RNA-binding proteins upon mTOR inactivation. Representative western blots of proteins changing their RNA binding following mTOR inhibition from independent oligo(dT) pulldowns *n* = 5.

We carried out a time-course to establish the optimal conditions under which both mTORC1 and mTORC2 are inhibited by Torin1 in HeLa cells (Supplementary Figure S1A). Consistent with dual inhibition of mTORC1 and mTORC2 function, we observed decreased phosphorylation of AKT, p70S6K, RPS6 and 4E-BP1 (Supplementary Figure S1Ai) following 1 h exposure to the drug (17,69). eIF4F complex integrity was assessed by m^7^GTP-sepharose affinity chromatography. The association of eIF4E with eIF4G1 was markedly decreased after 1 h of drug treatment and was accompanied by a concurrent increase in the association of 4E-BP1 with eIF4E (Supplementary Figure S1Aii). Northern blot analysis of sucrose density gradients demonstrated that TOP-containing mRNAs including eEF2, RPS16 and RPL24 all shifted to the sub-polysomes (non-translating state) upon mTOR inhibition, as expected (70), with less of a change in the control β-actin transcript, a non-TOP mRNA (Figure 1A). This confirmed that the experimental conditions at this 1 h timepoint were optimal for further investigations.

To identify the RBPs that change their RNA binding capacity to polyA+ RNA following mTOR inhibition, we performed RNA interactome capture adapted from Castello *et al.* (71), as described in materials and methods, 1 h after exposure to Torin1 (Figure 1B, Supplementary Figure S1B (72)). Affinity purified RBPs (Supplementary Figure S1C) were identified and quantified by mass spectrometry and log fold change was plotted against log adjusted *P*-value (Figure 1C). Of the 214 RNA-binding proteins present in all three replicates (Supplementary Table S1), two proteins showed increased and 20 decreased binding to RNA following mTOR inhibition (Supplementary Table S2). These observations were validated by western blot analysis (Figure 1D); two proteins, HNRNPQ and PSF, which did not change their RNA binding following mTOR inhibition were used as positive controls (Figure 1D). Our data show that LARP1 increased its association to RNA following mTOR inhibition (Figure 1D). This protein has been implicated in TOP mRNA regulation (38–42,73–77), binding directly to the m^7^Gppp-C cap and the adjacent TOP motif *via* the DM15 region (39–40,42). Similarly, the E3 ubiquitin ligase TRIM25 (78–80) displayed increased binding following mTOR inhibition (Figure 1D, Supplementary Table S1). TRIM25 has been shown previously to interact with non-coding RNAs (81). RBPs that showed decreased RNA binding included the ribosome associated protein SERBP1 (PAIR-B) (82–84) and PWP2. PWP2 has known roles in pre-ribosomal RNA processing and ribosomal biogenesis (85,86) and has been linked to regulation by S6 kinases (87) (double knockout of S6K1 and S6K2 cause a decrease in transcript levels of PWP2). A number of ribosomal proteins, including ribosomal protein RPS10, decreased their binding to RNA subsequent to mTOR inhibition, indicative of translational repression (88). Whilst mTOR inhibition would also be consistent with certain eIF4F complex components including eIF4G decreasing in association (Supplementary Figure S1A), within the RNA interactome capture data we observed eIF4G showing a decrease in binding following mTOR inhibition, however this failed to reach significance (Supplementary Table S1).

To identify the RNAs bound by TRIM25 and LARP1 (increased RNA binding), and SERBP1 and PWP2 (decreased RNA binding), flag-tagged versions of each of these four proteins were transiently overexpressed in HeLa cells. RNA-immunoprecipitation (RIP) was performed (Supplementary Figure S2A) and the associated RNAs were extracted and analysed on gene expression microarrays. LARP1 showed enrichment of the greatest number of mRNAs, 2546 (Supplementary Figure S2B), followed by SERBP1 with 1261 mRNAs, whereas TRIM25 and PWP2 showed no significant mRNA enrichment consistent with their previously published roles in non-coding and ribosomal RNA interactions. A significant number of TOP mRNAs that encode ribosomal proteins were among the LARP1-enriched mRNAs (Supplementary Figure S2C).

### Only a subset of LARP1-bound mRNAs are dependent on mTOR

**Figure 2.**
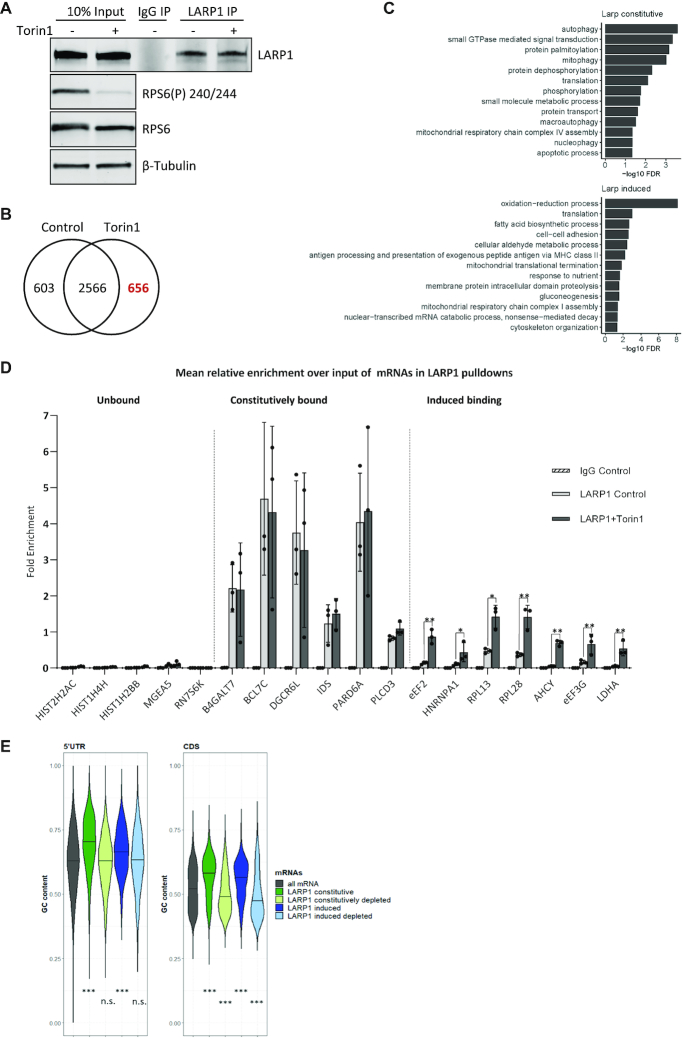
mRNAs associated with LARP1 with and without mTOR inactivation. HeLa cells were serum stimulated followed by control or Torin1 treatment. Endogenous LARP1 immunoprecipitation was performed and beads split for analysis of RNA and proteins. (A–C) mRNA arrays were performed using RNA extracted from both input and LARP1 pulldowns in control and mTOR inhibited conditions. The experiment was performed four times independently. (**A**) Western blots of endogenous LARP1 immunoprecipitates and 10% input samples from one representative experiment using indicated antibodies. (**B**) Number of RNAs significantly enriched over input in LARP1 IPs (Control log FC > 0.5 & Torin1 log FC > 0.5). (**C**) Genes uniquely assigned to either the induced or constitutively bound groups were assessed for enrichment of gene ontology biological process (BP) terms using David functional annotation. The graphs present terms enriched with an FDR < 0.05. (**D**) qPCR validation of RNA enrichment over input using primers for indicated RNAs, expressed as mean relative enrichment in LARP1 pulldown over input. mRNAs defined as unbound, constitutively bound or induced bound to LARP1 as described in main text. Paired 2-tailed t test on the means of three independent experiments. (**E**) Analysis of the GC content of 5′UTR and CDS sequences of all RefSeq mRNAs and LARP1 bound mRNAs. The term constitutively depleted refers to mRNAs that are significantly depleted (LogFC > –0.5) in both control and mTOR inhibited conditions and ‘induced depleted’ refers to mRNAs that are unbound in control conditions (FDR > 0.05) and depleted (log FC < –0.5) in Torin1-treated conditions. Medians indicated by black lines and statistical significance calculated using Kruskal-Wallis followed by Dunn test.

To determine the effect of mTOR inhibition on LARP1-dependent mRNA binding, we performed RNA-IPs (RIP) of endogenous LARP1 from HeLa cells ± mTOR inhibition (Figure 2A). Microarray analysis of the associated mRNAs showed a large set of 2566 transcripts were bound by LARP1 in both conditions, henceforth referred to as constitutively bound, as well as 656 mRNAs with significantly increased binding following mTOR inhibition, henceforth referred to as having induced binding (Figure 2B and Supplementary Table S3). mRNAs with induced LARP1 binding after mTOR inhibition include the GO functional annotations for translation and response to nutrients (Figure 2C). The microarray results were validated by RT-qPCR (Figure 2D) and confirmed to be maintained and specific, not a result of post-lysis interactions (89) by performing UV cross-linking prior to RIP (Supplementary Figure S3). We confirmed that RNAs including histones such as Histone 2H2AC and non-coding RNAs such as RN7SK are not enriched in the LARP1 IP (Figure 2D); histone mRNAs are not regulated by mTOR (31) and, in general, do not have poly(A) tails (reviewed in (90)). Importantly, changes in RNA binding were not accompanied by significant changes in RNA levels following mTOR inhibition (Supplementary Figure S4). Detailed transcript analysis at the nucleoside-level reveals that the set of mRNAs bound by LARP1 (mTOR inhibition induced and constitutively bound mRNAs) have high GC content in both their coding sequence and 5′UTR (Figure 2E). Interestingly, within the coding region it is specifically the GC content at the third or ‘wobble’ position of codons that most differentiates LARP1 bound mRNAs (Supplementary Figure S5).

### LARP1 represses translation of TOP mRNAs downstream of mTOR

**Figure 3.**
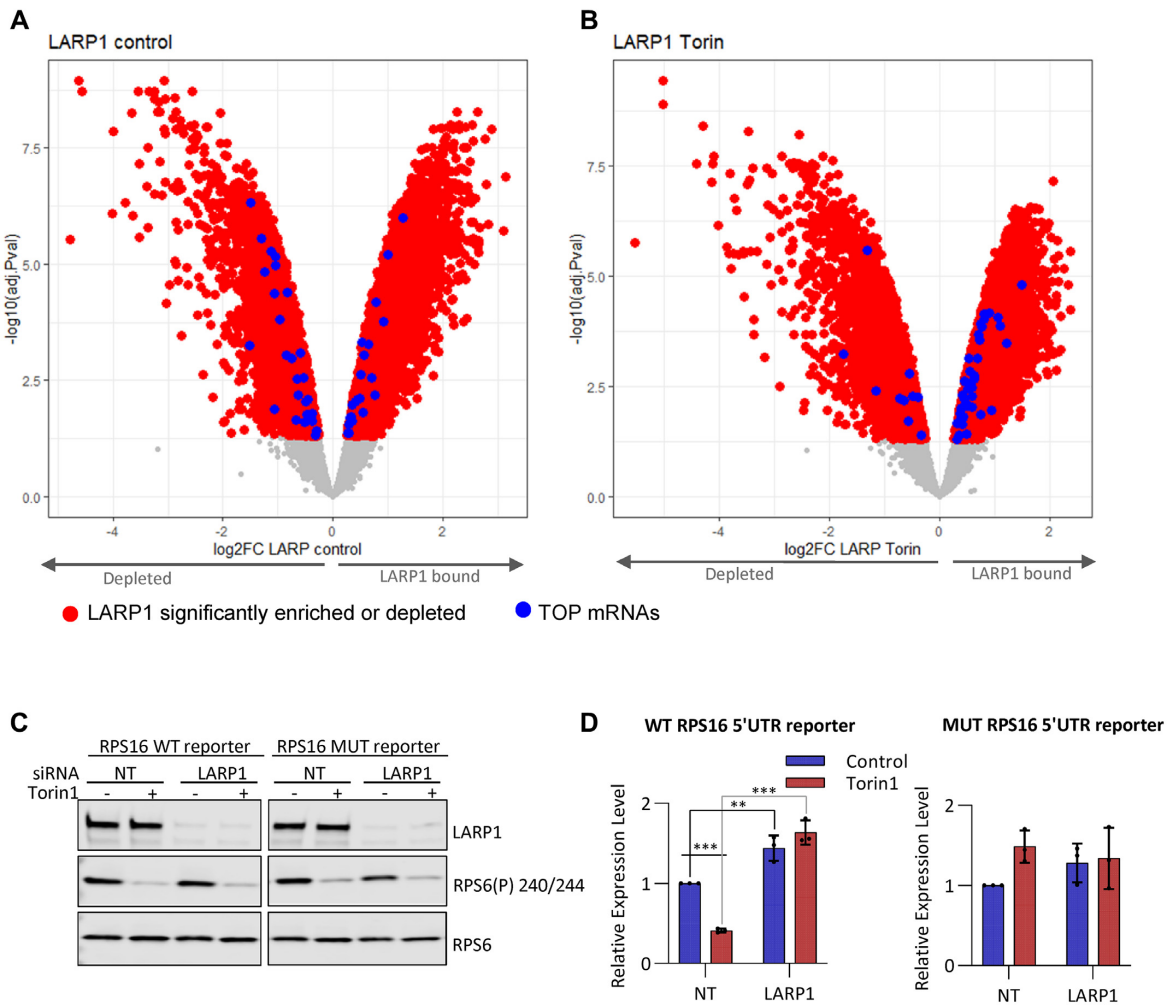
LARP1 binding to terminal oligopyrimidine tract containing mRNAs increases after mTOR inactivation. Log fold enrichment over input of all LARP1 bound mRNAs following immunoprecipitation from (**A**) Control treated or (**B**) Torin1 treated HeLa cells. mRNAs significantly (FDR < 0.05) enriched (LARP1 bound) or depleted over input shown in red, of these, blue data points represent TOP containing mRNAs as listed in (5). Non-significantly enriched mRNAs are in grey. C and D: HeLa cells transfected at a final concentration of 20 nM control or LARP1 siRNA were subsequently transfected with RPS16- PEST-CL reporters and control pRL-SV40 vector (Promega E2231). After 24 h, cells were treated with Torin1 or DMSO (control) for 2h followed by lysis. Experiment performed three times. (**C**) Western blots were performed using indicated antibodies. Representative western blots shown from one experiment of three. (**D**) Luciferase assays were performed in duplicate for each of 3 triplicate wells. Relative luciferase activity was calculated as a ratio of firefly luciferase to renilla luciferase. These were then normalized to firefly RNA levels to determine luciferase protein expression changes. The means and individual data points of three independent experiments are plotted with error bars indicating SD. Non-RNA normalized data is shown in Supplementary Figure S6.

As mentioned earlier, the mRNAs with increased association with LARP1 following mTOR inhibition include GO terms relating to translation and response to nutrients (Figure 2C). Consistent with this and in agreement with earlier studies including LARP1-CLIP experiments (38–40,73), we find enrichment of TOP mRNAs with LARP1 after mTOR inhibition (Figure 3A and B). We did not find a large overlap between our LARP1 RIP data and the CLIP data from Hong *et al* (73), possibly due to the distinct methodologies and biological systems employed. To further understand the role of LARP1 on the expression of TOP mRNAs, luciferase reporter constructs containing the promoter and 5′UTR of RPS16 upstream of firefly luciferase were used (further vector details in materials and methods). A vector containing five mutations within the first six bases of the TOP motif (CCUUUUCC mutated to GAGUGACC) was employed as a mutated-TOP negative control (Figure 3C, D and Supplementary Figure S6). Luciferase activity was examined in control and LARP1 knockdown conditions +/- Torin1 treatment ((Figure 3D and Supplementary Figure S6). We observed that, in the presence of LARP1, the WT RPS16 5′UTR-containing reporter, but not the mutant, was susceptible to mTOR inhibition and that this is dependent on LARP1, as its depletion abrogated the reporter's sensitivity to mTOR inhibition ((Figure 3C, D, Supplementary Figure S6).

### Translational control of LARP1 bound mRNAs

**Figure 4.**
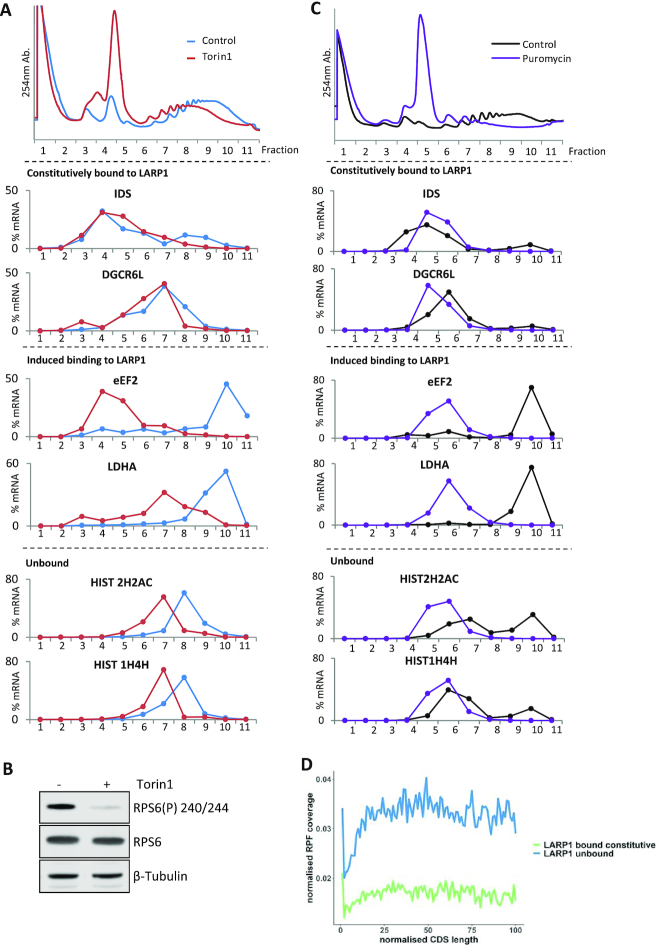
Polysome distribution of LARP1 bound mRNAs. HeLa cells supplemented with serum and then 200 nM Torin1 or DMSO vehicle control. (**A-C**) Sucrose density gradients were run and RNAs extracted from individual fractions. (**A**) Top panel: UV 254 traces of polysome gradients from control and Torin1 treated HeLa cell extracts. Lower panels: Percentage mRNA distribution across polysome gradients calculated from RT-qPCR. Data presented from one experiment representative of three independent experiments. (**B**) Western blots of input samples from A, for one of three independent experiments using indicated antibodies. (**C**) HeLa cells were treated with 200 μg/ml Puromycin for 1 h or 100 μg/ml cycloheximide for 3 min (Control). Sucrose gradients as in A. RNA was extracted from individual fractions and qPCR performed. Top panel: UV 254 traces of polysome gradients from control and puromycin treated HeLa cell extracts. Lower panels: Representative percentage mRNA distribution across polysome gradients calculated from RT-qPCR. (**D**) LARP1 constitutively bound mRNAs show reduced ribosome occupancy across the CDS. Graph shows RPF coverage normalized for mRNA abundance against normalized CDS length.

To probe the relationship between LARP1 binding and translation on endogenous mRNAs, HeLa cells ± Torin1 treatment were subjected to sucrose gradient analysis (Figure 4A). The distribution of several LARP1-bound mRNAs across the polysome gradients was measured by RT-qPCR (Figure 4A). mRNAs that showed increased binding to LARP1 following mTOR inhibition, including eEF2 and LDHA, relocated from a ‘heavy’ polysomal distribution (containing many ribosomes) to a ‘light’ (containing 2–4 ribosomes) or sub-polysomal distribution (Figure 4A and Supplementary Figure S7A), indicative of translational inhibition. Interestingly, the constitutively-bound mRNAs displayed a distinct distribution across the gradients. Firstly, they appear to be loaded with fewer ribosomes and secondly, show no net change in polysome association following mTOR inhibition (Figure 4A, B and Supplementary Figure S7A). To assess if these mRNAs were translationally stalled/silenced, cells were treated with puromycin to dissociate actively elongating ribosomes. The data show that the distribution of mRNAs with induced binding to LARP1 following mTOR inhibition relocated to lighter fractions of the gradient following puromycin treatment. However, mRNAs that bind to LARP1 irrespective of mTOR inhibition, do not shift to lighter polysomes after puromycin treatment (Figure 4C, right hand panel and Supplementary Figure S7B). The puromycin insensitivity suggests that the majority of these mRNAs are not actively engaged with translocating ribosomes, but they are presumably associated with as yet undefined RNP complexes (91). To further examine if the LARP1-associated mRNAs are globally translationally repressed and whether repression is dependent upon LARP1, a pre-existing ribosome profiling dataset from Philippe et al (43) was interrogated. This study included ribosome profiling in HEK293 cell lines with and without LARP1, ± Torin1 treatment. We examined the groups of mRNAs that were either unbound, constitutively or induced bound following mTOR inhibition. Density plots of translational efficiency (TE) for these groups is shown (Supplementary Figure S8) for HEK293 cells with LARP1 knockout (KO) (dark and light blue) in comparison to the control wild type (WT) cell line (dark and light green). The mean translational efficiency (⁞) of both LARP1 constitutively bound mRNAs and LARP1 mTOR inhibition induced bound mRNAs increases in the absence of LARP1. Whereas mRNAs that are not bound to LARP1 show no change in translational efficiency in the presence or absence of LARP1 (bottom panel). This indicates that LARP1 represses the translation of associated mRNAs. In addition, this data also shows that mRNAs bound by LARP1 following mTOR inhibition (LARP1 induced bound mRNAs) show a decrease in TE following Torin1 treatment in control cells (middle panel, dark green compared to light green) and this is slightly reduced in the knockout cells (dark blue compared to light blue). However, the TE of the constitutively bound mRNAs is not majorly affected by mTOR inhibition (top panel). Importantly, a metagene analysis showed that the mean ribosome occupancy across transcript open reading frames for LARP1 constitutively bound mRNAs is lower than unbound mRNAs (Figure 4D). Altogether, these data suggest that LARP1 binding correlates with reduced ribosomal occupancy—i.e. LARP1 likely represses the translation of mTOR-sensitive and importantly insensitive mRNAs.

### PABPC1-bound RNA changes following mTOR inhibition overlap strongly with LARP1

LARP1 has been previously shown to interact with PABPC1 directly (38,92), and it was therefore important to assess how PABP influences the binding of LARP1 to specific mRNAs, particularly as PABPC1 has been shown previously to activate translation (93,94). Endogenous LARP1 pulldowns in the presence of RNase I confirmed an RNA-independent interaction with PABPC1, which is unaffected by mTOR inhibition (Supplementary Figure S9A–C). We also tested whether bacterially expressed LARP1 could interact with PABPC1 and found that these two proteins did interact (Supplementary Figure S9D) and the amount of PABPC1 co-precipitating in His-LARP1 pulldowns increased with the addition of Dye680-labelled A20 RNA. This suggests that the interaction of LARP1 and PABPC1 may be strengthened by the association with RNA.

**Figure 5.**
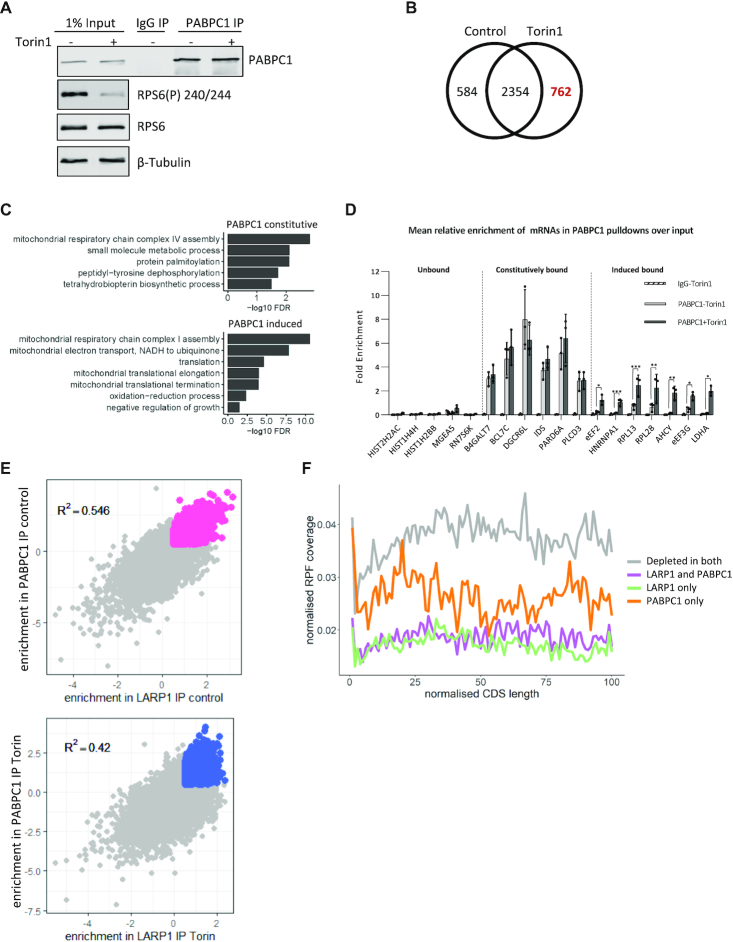
mRNAs associated with endogenous PABPC1 with/without mTOR inactivation and overlap with LARP1. (A–D) PABPC1 and control IgG immunoprecipitation from HeLa cells following mTOR inactivation. (**A**) Representative western blots of PABPC1 IP and input samples using indicated antibodies. (**B**) RNAs significantly enriched over input (log FC > 0.5) in PABPC1 IPs. Analysis performed as in Figure 2. (**C**) Genes uniquely assigned to either the induced following mTOR inhibition or constitutive group for PABPC1 were assessed for enrichment of gene ontology biological process (BP) terms using David functional annotation. The graphs present terms enriched with an FDR < 0.05. (**D**) QPCR validation of RNA binding to PABPC1 following mTOR inhibition. (**E**) Overlap of RNA enrichment over input in PABPC1 and LARP1 IPs from either control or Torin1 (mTOR inhibited) conditions. Pink and blue dots represent statistically significant enriched mRNAs in overlapped IPs from control or Torin1 conditions, respectively. Gray dots are non-significantly enriched mRNAs. R^2^ values displayed. (**F**) mRNAs bound by LARP1 or LARP1 & PABPC1 display decreased ribosome occupancy throughout the CDS. Groups used for this figure are listed in Supplementary Table S7.

We hypothesized that the PABPC1-LARP1 interaction may be involved in the differential sensitivity of LARP1-bound mRNAs to mTOR. To examine the role of PABPC1 in this regard, PABPC1 RNA-IP was carried out ±mTOR inhibition and the mRNAs analysed by cDNA microarray (Figure 5A), an experiment complementary to the LARP1 RIP (Figure 2). The data show (Figure 5B and Supplementary Table S3) PABPC1 bound to 3700 mRNAs, a large number, which was unsurprising given its role in mRNA translation (93,94). Gene ontology enrichment analysis showed that upon mTOR inhibition there was an enrichment of mRNAs encoding proteins related to translation and mitochondrial translational elongation/termination (Figure 5C). RT-qPCR validation showed that the mRNAs bound by PABPC1 have a similar profile to that of LARP1, with two distinct groups of mRNAs identified: those constitutively bound by PABPC1 and those where mTOR inhibition increased mRNAs binding (Figure 5D). We then examined the degree of overlap of mRNAs bound to both LARP1 and PABPC1 in untreated cells or following mTOR inhibition (Figure 5E). The data show that there is a strong positive correlation in enrichment of mRNAs recovered in the LARP1/PABPC1 immunoprecipitations in both the control and Torin1 treated cells. The extent of the overlap was somewhat surprising given that PABPC1 has a well-described function as a positive regulator of translation. Indeed, examining ribosome profiling data for the ribosome occupancy across the coding sequences of groups of mRNAs bound by LARP1 and PABPC1, we observe that the mean ribosome occupancy of LARP1 and LARP1/PABPC1 bound mRNAs is lower than of those bound to PABPC1 alone (presumably when associated with the eIF4F complex), supporting a role of PABPC1 as an activator of translation when independent of LARP1 (Figure 5F, RNA groupings are defined in Supplementary Table S7). The PABPC1 bound mRNAs (orange) show lower mean ribosome occupancy than depleted mRNAs (gray), which would indicate that they are translated less than the unbound mRNAs. There are several reasons why this may be the case. One is that many of the unbound mRNAs are non-canonical (non polyadenylated for example) transcripts such as histones and may generally be highly expressed and be regulated in a different manner from PABPC1 bound mRNAs. Secondly, PABPC1 associates with other regulatory proteins other than eIF4G, for example PAIP1 or PAIP2 and this may result in reducing the TE of this group. However, importantly, it appears to be the minority of mRNAs (264) that are bound to PABPC1 only, while the majority, 1010 mRNAs are PABPC1-LARP1 bound.

When we look at the translational efficiency of PABPC1–LARP1 bound mRNAs using TE data from Philippe *et al.* (43) (Supplementary Figure S10), mRNAs bound by both LARP1 and PABPC1 show increased TE in LARP1 knockout cells (dark and light green) compared to the same treatments in the control cell line (dark and light blue). LARP1 only bound mRNAs also have increased translational efficiency in LARP1 knockout cells. The TE of unbound mRNAs (third panel) show no difference between LARP1 knockout cells and controls. PABPC1 bound mRNAs show decreased TE following Torin1 treatment in control cells (bottom panel, dark green compared to light green) and this is slightly reduced in the knockout cells (light blue compared to dark blue). PABPC1 only bound mRNAs also show increased translational efficiency in LARP1 knockout cells, this could in part be due to some of these mRNAs being bound and controlled in some instances by LARP1 at lower levels than our threshold cut-off for binding/enrichment. Overall, this data shows that LARP1–PABPC1 bound mRNAs are translated less efficiently than mRNAs bound to PABPC1 only or unbound mRNAs.

The extent of PABPC1 binding to mRNA is strongly influenced by poly (A) tail length (95). However, poly(A)-tail length (PAT) assays showed that there were no gross changes in the poly(A) tail length of mRNAs from LARP1 bound or unbound groups during this time window of Torin1 treatment (Supplementary Figure S11).

### Preventing LARP1/PABPC1 interaction severely disrupts LARP1 mRNA interaction

**Figure 6.**
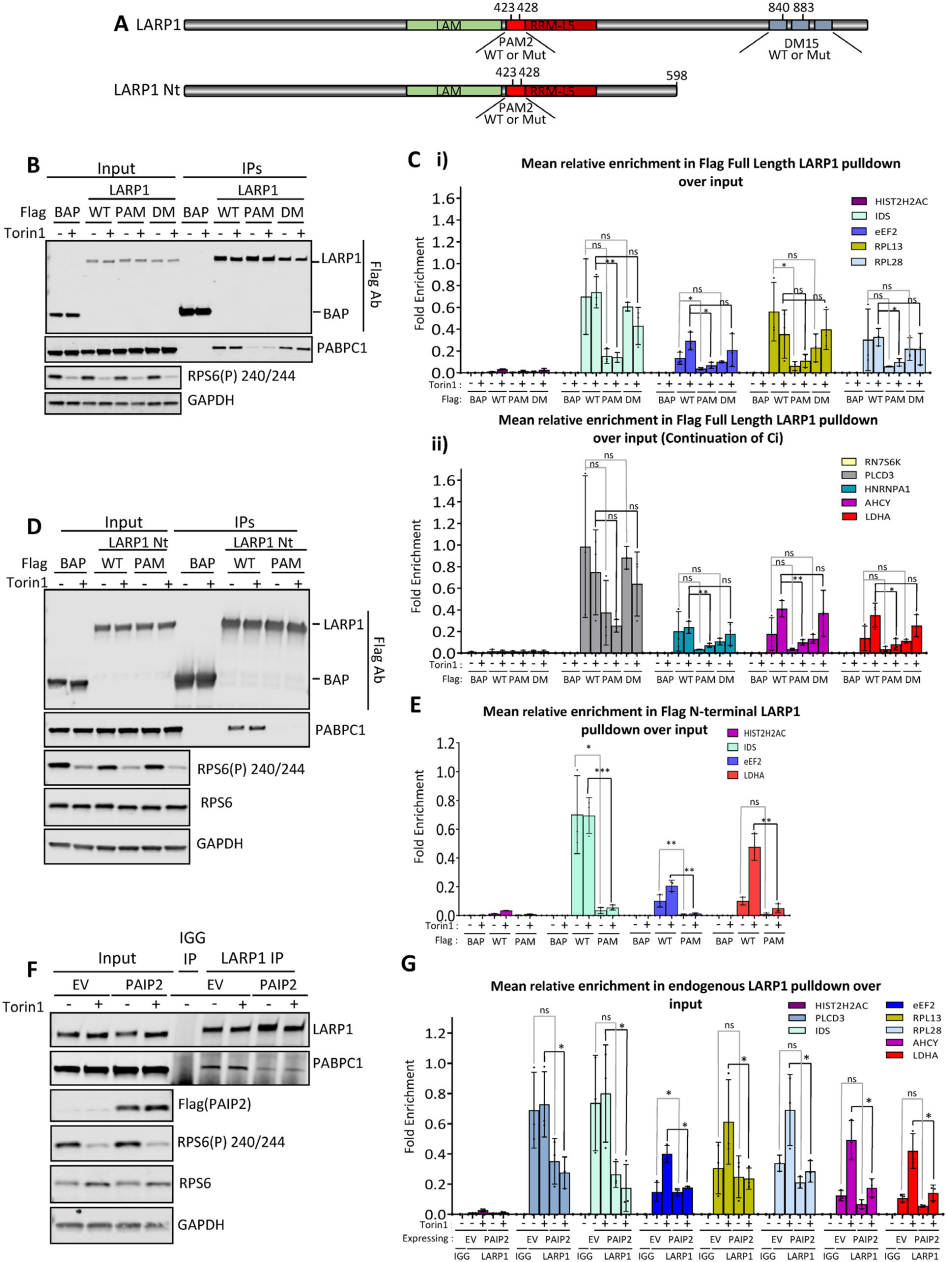
Dissecting the functional domains of LARP1. (**A**) Schematic representation of the LARP1 constructs used in overexpression experiments. Amino acid numbering based on LARP1 Isoform 2 (Q6PKG0–3) B-C. Immunoprecipitation of transiently overexpressed Flag-tagged LARP1 2–1019: wild type (WT); PAM2 L423A/F428A double point mutant (PAM); DM15 R840E/Y883A double point mutant (DM) or flag tagged bacterial alkaline phosphatase (BAP) control protein from HeLa cells treated with or without Torin1. (**B**) Western blots of Flag tagged protein overexpression in 10% inputs and immunoprecipitations from one representative experiment of three. (**C**) (**i**) and (**ii**). Mean relative enrichment from qPCR analysis as mean relative enrichment in Flag pulldowns over input with data points from three individual experiments plotted. D-E. HeLa cells were transiently transfected with either: Flag tagged wild type LARP1 N-terminus (‘WT LARP1 Nt’ -amino acids 2–598) wild type (WT); LARP1 N-terminus PAM2 L423A/F428A double point mutant (PAM) or bacterial alkaline phosphatase (BAP) flag tagged control protein. Cells were treated plus or minus mTOR inhibition using Torin1 and flag immunoprecipitations performed. (**D**) Western blots of Flag tagged LARP1 N-terminal (Nt) (2–598) truncation overexpression in 10% inputs and immunoprecipitations using indicated antisera. Blots are from one representative experiment, *N* = 3. (**E**) Mean relative enrichment from qPCR as in C. (F and G) HeLa cells were transiently transfected with empty vector or PAIP2 and left for 24h. Endogenous LARP1 immunoprecipitations were then performed from control or 200 nM Torin1 treated cells. (**F**) Westerns blots showing mTOR inhibition and overexpression of PAIP2 and the resulting reduced capacity of PABPC1 to bind to LARP1 using indicated antibodies. Western blots from one representative experiment, *N* = 3. (**G**) qPCR enrichment analysis of LARP1 bound mRNAs in the absence and presence of PAIP2 overexpression as in (C).

LARP1 possesses several RNA binding motifs, including within the La module which has been reported to bind poly(A) and pyrimidine rich mRNA (77); a conserved La motif (LAM) and the RRM5-like RNA binding domain. The La module additionally contains a PAM2 (PABPC1-binding) domain (38,96) (Figure 6A). There is scope for the LAM and RRM-L5 to act synergistically with regards to substrate binding as found in other La protein family members (97), however for LARP1 this has not been fully investigated. Mutation of the PAM2 domain has been previously shown to reduce PABP binding (38). The DM15 region (comprised of three DM15 modules) is unique to LARP1/LARP1B and lies towards the C-terminus of the protein (40).

Given that our data suggest that LARP1 and PABPC1 bind similarly to mRNAs that do or do not change their interaction following mTOR inhibition, we investigated the relative contributions of the PAM2 and DM15 domains to these interactions.

To assess the relative impact of LARP1′s interaction with both PABPC1 via the PAM2 domain or directly with mRNA via the DM15 domain, mutations were introduced into these domains of LARP1. Flag-tagged version of LARP1 wild type (WT) and versions containing point mutations in two conserved residues of the PAM2 domain (L423A/F428A, Figure 6A (38)) or two residues in the DM15 domain of LARP1 (R840 and Y883), which have been shown previously to be required for TOP mRNA binding and cap structure recognition, respectively (39,40) were overexpressed. Flag affinity purifications of LARP1 wild type (WT) and mutants were performed (Figure 6B) and associated RNAs were extracted, with flag-bacterial alkaline phosphatase (BAP) used as a negative control. As expected, the mutations in the PAM2 domain resulted in a decreased PABPC1–LARP1 interaction, but unexpectedly this correlated with a significant decrease in overall RNA binding (Figure 6Ci, ii and Supplementary Figure S12A). A similar decrease was also observed when using Flag-tagged N-terminal LARP1 containing the PAM2 L423A/F428A mutations (Figure 6D and E). Interestingly, in the context of full length LARP1, the DM15 R840E/Y883A mutations did not cause any significant decrease in the capacity of LARP1 to bind RNA, including the TOP-containing mRNAs, eEF2, RPL13, RPL28 and HNRNPA1 (Figure 6Ci and ii), nor did they have an effect on PABPC1 binding (Figure 6B). The sustained mRNA binding may in part be due to the La module mRNA binding (77). We however confirm that the C-terminal 615–1019 section of LARP1 (Supplementary Figure S13A–C) shows enhanced binding to LARP1 target mRNAs in comparison to BAP control and that these interactions are perturbed by the R840E/Y883A mutations (Supplementary Figure S13C). While this demonstrated the importance of these residues for DM15-mRNA interactions, the C-terminal portion of LARP1 did not confer the mRNA binding specificity observed with wildtype protein, with histone 2H2AC mRNA now interacting (Supplementary Figure S13C, Figure 6C). This could mean that either PABPC1 or other RNA binding domains in LARP1′s N-terminus direct LARP1 to specific mRNAs. The role of the PAM2 domain and the interaction with PABPC1 was also highlighted by the observation that the LARP1 L423A/F428A mutant did not associate with polysomes in comparison to wild type LARP1 (Supplementary Figure S14).

The LARP1 La module has previously been shown to bind both poly(A) and CU rich mRNA by Al-Ashtal *et al.* (77). To address the question whether PABPC1 is required for the delivery of these mRNAs to LARP1 using a distinct experimental approach, we over-expressed the PABPC1 binding protein PAIP2, which interacts with the MLLE domain of PABPC1 via its PAM2 domain (Figure 6F). As expected, this resulted in the sequestration of PABPC1, prevented its interaction with LARP1 (Figure 6F) and this resulted in a decrease of RNA association with LARP1 of a panel of mRNAs (Figure 6G and Supplementary Figure S12B). These data suggest that PABPC1 directs or induces a conformational change in LARP1 required for increased RNA binding. Our findings also provide further rationale for the specificity of LARP1 to poly(A) tail-containing mRNAs supported by the data which show that histone mRNAs, which lack poly(A) tails (98), are not bound by LARP1.

## DISCUSSION

RNA-binding protein capture was used to identify the changes in the RNA protein interaction network following mTOR inhibition (Figure 1 and Supplementary Tables S1 and S2). In total, we identified 22 proteins that change their association with RNA following mTOR inhibition, two of which, LARP1 and TRIM25, increased their binding to mRNA. LARP1 has been implicated in the regulation of TOP mRNAs downstream of mTOR (38–41,73,75,77) and our data confirm this. This activity may be context dependent since other data demonstrate that insulin (a stimulator of mTOR signalling, reviewed in (99)) increases the binding of LARP1 to TOP-containing mRNAs (41). Here, we find that endogenous LARP1 associates with TOP mRNAs, in addition to a large subset of other mRNAs (Figures 2 and 3), and that this binding correlates with their translational repression. The E3 ubiquitin ligase TRIM25 was also identified as increasing its RNA binding following mTOR inhibition. TRIM25′s ubiquitin ligase activity has been reported to be stimulated by RNA binding (81). The 20 proteins which reduced their interaction with mRNA following mTOR inhibition (Figure 1C and Supplementary Table S2), included ribosomal proteins and associated factors, e.g. SERBP1 (82–84) and these are currently under further investigation.

We identified nearly 4000 mRNAs that are enriched in LARP1 immunoprecipitates (Figure 2B), and these formed two distinct groups, i.e. mRNAs that change their association with LARP1 following mTOR inhibition (referred to as ‘induced’) and those bound to LARP1 under both conditions (‘constitutively’ bound). Importantly, our data confirmed that LARP1 can interact directly with PABPC1 (Supplementary Figure S9) and examination of the mRNAs that associated with both LARP1 and PABPC1 suggested that they are co-ordinately regulated by these proteins following mTOR inhibition (Figure 5E). Recent work on LARP1 has highlighted that LARP1 plays a role in the regulation of mRNAs important for mitochondrial function (100) and work by Zhang has shown that Drosophila LARP shows some mitochondrial outer membrane localization (101) where it controls the translation of cytoplasmic mRNAs important for mitochondrial function. Additionally, studies have now shown that PINK1 phosphorylation of LARP represses local translation at the mitochondrial surface (102). Indeed we see mitochondrial related GO terms in both the LARP1 and PABPC1 RIP analysis, especially with PABPC1 following mTOR inhibition (Figures 2C and 5C respectively). The bottom two panels of Supplementary Figure S7A show polysome distribution of two nuclear-derived mRNAs that encode mitochondrial proteins that exhibited increased binding after mTOR inhibition to both PABPC1 and LARP1 (mRNAs encoding the mitochondrial acyl carrier protein NDUFB5 and FARS2, a mitochondrial tRNA ligase). We find that these two mRNAs come out of the polysomes following mTOR inhibition (Supplementary Figure S7A) suggesting decreased translation upon mTOR inhibition.

We show that the PAM2 domain of LARP1 is required for its interaction with PABPC1 (Figure 6) and importantly that mutation of the PAM2 domain or overexpression of PAIP2, which sequesters PABPC1 away from LARP1, result not only in the loss of PABPC1 association, but also results in a large reduction in specific mRNA binding (Figure 6). Overall, our data suggest that mRNA specificity is driven by the LARP1-PABPC1 interaction (Figure 6 and Supplementary Figure S13C) and that in the context of the full length protein the DM15 region of LARP1 does not impact on its interaction with PABPC1 and associated mRNAs (Figure 5B-C). While the mutations in the DM15 domain do not result in loss of mRNA association it would be expected that lack of cap-binding may prevent mRNA circularization (Supplementary Figure S15). Multivalent interactions (interactions involving several interaction points) can influence and produce cooperative and sensitive binding dynamics and thus enhance the specificity and or functional affinity. The association of PABPC1 and LARP1 to each other (Supplemental Figure S9) and to the same RNA cohorts (Figure 5E) along with the data that disruption of this interaction (Figure 6F and G) reduces RNA binding suggest that multivalency may well be playing a role in the dynamics of LARP1-PABPC1-RNA interaction.

The translational efficiency of LARP1-bound mRNAs is lower than RNAs not enriched on LARP1 (Figure 4D, Supplementary Figure S8). Importantly, mRNAs bound by PABPC1, but not LARP1, have higher translational efficiency than LARP1- and LARP1/PABPC1-bound mRNAs (Figure 5F). These results indicate that a subset of mRNAs bound by PABPC1 but LARP1-independent are highly translated, presumably eIF4F-associated. Given that LARP1 can outcompete eIF4E for binding to C-capped mRNA and that LARP1 binds to PABPC1, we hypothesize that the LARP1/PABPC1 interaction allows for an eIF4E-independent closed loop conformation on mRNAs with induced binding following mTOR inhibition (Supplementary Figure S15).

It is not clear how the mRNAs that are constitutively bound by LARP1 and PABPC1 are translationally repressed by these proteins, or whether this inhibition is regulated downstream of different signalling events or stimuli. One possibility is that when LARP1 and PABPC1 bind to a single transcript, the mRNA, in addition to being translationally repressed is also stabilized *via* protection from deadenylation by PABPC1 and de-capping by LARP1 *via* binding to the 5′ end of the transcript through the DM15 domain.

Taken together, our findings suggest that LARP1 is a negative regulator of translation through its interaction with PABPC1 and this interaction is key for targeting LARP1 to specific mRNA. Perturbation of this interaction disrupts LARP1 mRNA binding. While our data in this context confirms LARP1 to be a translational repressor, we cannot exclude that its activity is context dependent.

## DATA AVAILABILITY

The mass spectrometry proteomics data has been deposited to the ProteomeXchange Consortium (http://proteomecentral.proteomexchange.org) *via* the PRIDE partner repository (103) with the dataset identifier PXD016705 and DOI 10.6019/PXD016705.

Array data discussed in this publication has been deposited in NCBI’s Gene Expression Omnibus (104) and are accessible through GEO Series accession number GSE142539. (https://www.ncbi.nlm.nih.gov/geo/query/acc.cgi?acc=GSE142539).

## Supplementary Material

gkaa1189_Supplemental_Files
