# Supplementary material for: The mTOR regulated RNA-binding protein LARP1 requires PABPC1 for guided mRNA interaction
Source: Nucleic Acids Res. 2020 Dec 17;49(1):458–78. doi: 10.1093/nar/gkaa1189 (PMC7797073; doi:10.1093/nar/gkaa1189)
Supplement: gkaa1189_Supplemental_Files [file gkaa1189_supplemental_files.zip › Supplementary Data.pdf]

SUPPLEMENTARY DATA

SUPPLEMENTAL FIGURE AND TABLE LEGENDS

Supplementary Figure S1. Time course of mTOR inhibition and oligo(dT) pulldown optimisation. A. Time course of mTOR inactivation using 200 nM Torin1 on HeLa cells. **i)** Western blots on lysates and **ii)** m^7GTP (cap analogue) pulldowns from each time point with indicated antibodies to assess 4E-BP1, eIF4G and eIF4E association as indicated. Representative blots from one of 3 independent experiments. **B.** Oligo(dT) interactome capture pulldowns from HeLa cells **i)** Silver stain gel of control (-) versus UV254 cross-linked (CL) inputs and oligo(dT) pulldowns from a representative experiment. **ii)** Western blots of control versus UV254 cross-linked inputs and oligo(dT) pulldowns using indicated antisera. **C.** Coomassie stained gel of control (DMSO vehicle) and Torin1 treated oligo(dT) pulldown samples used for mass spectrometry (Figure 1B and C). Representative of three independent experiments.

Supplementary Figure S2. RNA binding protein pulldown and microarray. Hela cells were transiently transfected with Flag-tagged GFP, LARP1, TRIM25, SERBP1 or PWP2, and then subsequently treated with 200 nM Torin1 or DMSO control. Cells were lysed and flag pulldowns performed as described in materials and methods. Two independent experiments were performed **A.** 10% of input and flag pulldowns were subjected to SDS-PAGE and western blotting with the indicated antibodies. **B-C**. RNA was extracted from input and pulldowns and prepared for gene expression arrays. **B.** Table showing the number of RNAs significantly enriched over input for the four Flag tagged RNA binding proteins individually. **C.** Volcano plot of mRNA enrichment over input from Flag LARP1 pulldowns. mRNAs significantly enriched or depleted over input shown in red, of these, ribosomal protein mRNAs plotted in blue. Non-significant mRNAs in grey.

Supplementary Figure S3. mRNAs associated with LARP1 with/without mTOR inactivation in the presence of UV254 cross-linking. Endogenous LARP1 immunoprecipitations were performed from control or 200 nM Torin1 treated HeLa cells after UV254 cross-linking (as described in Supplemental materials and methods). Three independent experiments performed. **A.** Representative western blots from endogenous LARP1 immunoprecipitations and 10% input samples using indicated antibodies. **B.** RT-qPCR was performed on purified RNA from input and IP samples using intron-spanning primers. Data plotted as mean relative enrichment in LARP1 pulldown over input. Data analysed using unpaired, two-tailed student’s t test. **C.** RT-qPCR on input RNA from the three experiments normalised to control.

Supplementary Figure S4. Input levels of mRNAs associated with LARP1 with/without mTOR inactivation. RT-qPCR relative input levels for RNAs shown in Figure 2D. Mean of 3 experiments with individual normalised data-points plotted.

Supplementary Figure S5. GC content of the wobble position is high in LARP1 bound mRNAs. Plots of GC content at codon positions 1 (GC1), 2 (GC2) and 3 (GC3) respectively from coding sequences of all mRNAs versus LARP1 bound or depleted mRNAs as indicated. Medians indicated by black lines and statistical significance calculated using Kruskal-Wallis followed by Dunn test.

Supplementary Figure S6. RPS16 TOP luciferase reporter assays in the presence of LARP1 knockdown -/+ mTOR inhibition. Luciferase assays from Figure 3D prior to normalization against firefly luciferase mRNA levels. Mean of 3 experiments normalised to control (NT) are plotted.

Supplementary Figure S7. Polysome distribution of LARP1 constitutively bound mRNAs does not change following mTOR inhibition. A. (Extended from Figure 4A). Representative percentage mRNA distribution across polysome gradients from HeLa cells +/- Torin1 treatment, from RT-qPCR of individual polysome fractions for LARP1 constitutively or mTOR inhibition induced bound mRNAs; groups as defined in Figure 2B-D and Supplementary Table S3. **B.** (Extended from Figure 4C). Polysome gradients from puromycin treated HeLa cells. Representative percentage mRNA distribution across polysome gradients calculated from RT-qPCR CT values from individual polysome fractions.

Supplementary Figure S8. mRNAs associated with LARP1 show increased translational efficiency in LARP1 knockout cells. Density plots of translational efficiency (TE) using RIboseq data from (1) of LARP1 RIP mRNA groupings: constitutively bound (larp_constitutive), mTOR inhibition induced bound (larp_induced) and unbound mRNAs are shown for each of four conditions: wild type (WT) or LARP1 single knockout (KO) HEK293T cells with either control or Torin1 treatment. The plots show that both LARP1 constitutively bound and mTOR inhibition induced bound mRNAs have increased TE in LARP1 knockout cells (dark and light green) compared to the same treatments in the control cell line (dark and light blue). Means shown as coloured dotted lines as indicated.

Supplementary Figure S9. The interaction of LARP1 and PABPC1 is RNA-independent. A-B. HeLa cells +/- Torin1 treatment were lysed in IP buffer in the presence of 150 U/ml RNase I and endogenous LARP1 was immunoprecipitated. **A.** Western blots from one of three experiments showing the interaction of PABPC1 with LARP1 immunoprecipitates in the absence of RNA. **B.** Agarose gel showing the loss of 18S and 28S ribosomal RNA in treated lysates to confirm RNA degradation. **C.** RNase I degradation assay. To investigate the activity of RNase I, specifically on an oligo-A sequence, 1 μM

Dye680-labelled A20 RNA was incubated with 150 U/ml RNase I in the presence of 8 µM competitor RNA for the reaction times indicated. Samples were subjected to TBE-urea gel electrophoresis and RNA bands were visualised. A dye680-labelled A2 dinucleotide was used as a size marker. Image from one of two independent experiments. **D**. Recombinant LARP1 and PABPC1 interact in vitro. Bacterially expressed His-LARP1 was incubated with bacterially expressed PABPC1 with/without Dye680-labelled A20-RNA. His or IgG immunoprecipitation was then carried out as indicated. Western blots were performed on input and IP with the indicated antibodies. A Coomassie stained gel (bottom panel) of immunoprecipitations and input samples is also shown (bottom panel). Images are from one of two independent experiments.

Supplementary Figure S10. mRNAs associated with LARP1 and PABP or LARP1 alone show increased translational efficiency in LARP1 knockout cells. Density plots of translational efficiency (TE) using RIboseq data from (1) split into the following RIP mRNA groupings: LARP1 and PABPC1: bound by both LARP1 and PABPC1 either constitutively or induced following mTOR inhibition; LARP1_only: either LARP1 constitutive or induced bound following mTOR inhibition, but not PABPC1 bound; PABPC1_only: either PABPC1 constitutive or induced bound mRNAs that are not bound by LARP1 and Neither; mRNAs not bound to LARP1 or PABPC1. TE are shown for each of four conditions: wild type (WT) or LARP1 knockout (KO) HEK293T cells with either control or Torin1 treatment. The plots show that mRNAs bound to both LARP1 and PABPC1 have increased TE in LARP1 knockout cells (dark and light green) compared to matched treatments in control cells (dark and light blue). Means shown as coloured dotted lines as indicated.

Supplementary Figure S11. Polyadenylation test shows no changes in poly(A) tail length following 1 h of mTOR inhibition on mRNAs bound by LARP1. HeLa cells were serum stimulated for 30 min followed by control or 200 nM Torin1 treatment for 1 hour. Extracted RNA was analysed by poly(A) test (PAT) for the six different targets indicated, representing genes that are either constitutively bound, induced upon mTOR inhibition or not bound to LARP1. Deadenylated RNA was generated by incubating total RNA with RNaseH and oligo(dT) where indicated. PAT PCR products were subjected to electrophoresis on 4% high resolution agarose gels (left panels) and signal distribution within each lane was quantified using ImageJ and plotted (right hand graphs).

Supplementary Figure S12. Affecting the LARP1/PABPC1 interaction disrupts LARP1 mRNA interaction. A. (Extended from Figure 6C). Mean relative enrichment qPCR analysis from Flag pulldowns with immunoprecipitation of transiently overexpressed Flag-tagged LARP1 2-1019: wild type (WT); PAM2 L423A/F428A double point mutant (PAM); DM15 R840E/Y883A double point mutant (DM) or flag tagged bacterial alkaline phosphatase (BAP) control protein from HeLa cells treated with

or without Torin1. Data points from three individual experiments plotted. **B.** (Extended data for Figure 6G). qPCR enrichment analysis of LARP1 bound mRNAs in the absence and presence of PAIP2 overexpression.

Supplementary Figure S13. The C-terminus of LARP1 requires the DM15 domains for mRNA binding but exhibits less RNA binding specificity than full length LARP1. Hela cells were transiently transfected with Flag tagged bacterial alkaline phosphatase (BAP), wild type (WT) C-terminal LARP1 (615-1019) or C-terminal LARP1 R840E/Y883A DM15 mutant (DM15M). 24h following transfection, cells were treated with DMSO (Control) or 200 nM Torin1. Cells were lysed and Flag pulldowns performed. **A.** Schematic of the C-terminal region (amino acids 615-1019) used in the overexpression and pulldown assays **B.** Representative western blots of Flag tagged protein overexpression within inputs and immunoprecipitation. Western blots of lysate to determine mTOR inhibition using indicated antibodies **C.** Mean relative enrichment in Flag pulldowns over input from RT-qPCR analysis using primers to indicated RNAs. Data analysed using two-tailed, two sample equal variance Student's t-test comparing wild type C-terminal region to DM15 double mutant between treatment matched samples. n=4 independent experiments.

Supplementary Figure S14. Localisation of exogenous LARP1 on polysome gradients is perturbed with PABPC1 binding mutant. HeLa cells were transiently transfected with 5 µg per plate Flag tagged LARP1 2-1019 wild type (WT) or PAM2 L423A/F428A double point mutant (PAM2M) constructs. 24h post transfection, control or 200 nM Torin1 treated cells were then lysed and gradient sedimentation, fractionation and western blotting performed as described in materials and methods.

Supplementary Figure S15. Model of LARP1 mRNA interaction. **A.** Under conditions where mTORC1 is active, it phosphorylates 4EBP1 and LARP1 through RAPTOR's interaction with these substrates. In these conditions LARP1 is bound to fewer mRNAs, but retains its PABPC1 binding. mRNAs bound to PABPC1 in the absence of LARP1 are actively translated. Upon Torin1 inactivation of mTOR, induced bound messages increase their 5'UTR binding (Hong et al (2)) and concomitantly PABPC1 increases its binding to these mRNAs. When mTOR is inactive and LARP1 binding increases, this correlates with translational repression. **B.** LARP1 binds to a large number of mRNAs that do not respond to mTOR activity fluctuations (constitutively bound mRNAs). These mRNAs are not heavily translating. It may be that the phosphorylation of LARP1 has no effect on the mRNA within the 3'UTR or CDS and only 5'UTR binding is affected by this phosphorylation (Hong et al (2)).

Supplementary Table S1. Table showing the 214 RNA binding proteins that were identified in the RNA binding protein screen (graphed in Figure 1C). Showing mean log fold change in enrichment following

mTOR inhibition, P. value and adjusted P. value (for RNAs graphed in Figure 1C). Fold change cut-off used 1.3FC, with adjusted p-values <0.2 (Benjamini-Hochberg).

Supplementary Table S2. Table showing mean log fold change in enrichment of RNA binding proteins that were significantly increased or decreased in their binding to RNA following mTOR inhibition (for RNAs graphed in Figure 1C). Fold change cut-off used 1.3FC, with adjusted p-values <0.2 (Benjamini-Hochberg).

Supplementary Table S3. Table showing mean log fold change enrichment of RNA immunoprecipitations for both LARP1 and PABPC1. For each of these two proteins individually, mRNAs considered to be enriched in the IP had a logFC > 0.5 and FDR < 0.05. RNAs binding to LARP1 or PABPC1 were divided into the following groups for subsequent analysis: "induced bound": logFC<=0 in control and logFC>0.5 in Torin1-treated; "constitutively bound": logFC> 0.5 in both control and Torin1-treated, "unbound": logFC< 0 FDR< 0.05 in both control and Torin1 conditions. For Figures 2E, Figure 5F and Supplementary Figure S5, to provide comparable groups to constitutively bound and induced bound mRNAs, unbound mRNAs were further broken down into "constitutively depleted": logFC< -0.5 in control and Torin1-treated and "induced depleted": FDR>0.05 in control and logFC< -0.5 in Torin1-treated.

Supplementary Table S4. List of qPCR primers and Northern probe-generation primers used in this study. (N) designates primers used for northern probe generation, all other primers were for RT-qPCR.

Supplementary Table S5. List of primers used for cloning and site-directed mutagenesis.

Supplementary Table S6. Polyadenylation Test Primers.

Supplementary Table S7. Fold change cut-offs for RNA groups used for subsequent analysis of LARP1 and PABPC1 bound mRNAs.

SUPPLEMENTARY REFERENCES

1. Philippe, L., van den Elzen, A.M.G., Watson, M.J. and Thoreen, C.C. (2020) Global analysis of LARP1 translation targets reveals tunable and dynamic features of 5' TOP motifs. *Proceedings of the National Academy of Sciences of the United States of America*, **117**, 5319-5328.
2. Hong, S., Freeberg, M.A., Han, T., Kamath, A., Yao, Y., Fukuda, T., Suzuki, T., Kim, J.K. and Inoki, K. (2017) LARP1 functions as a molecular switch for mTORC1-mediated translation of an essential class of mRNAs. *eLife*, **6**, e25237.

Supplementary Figure S1.

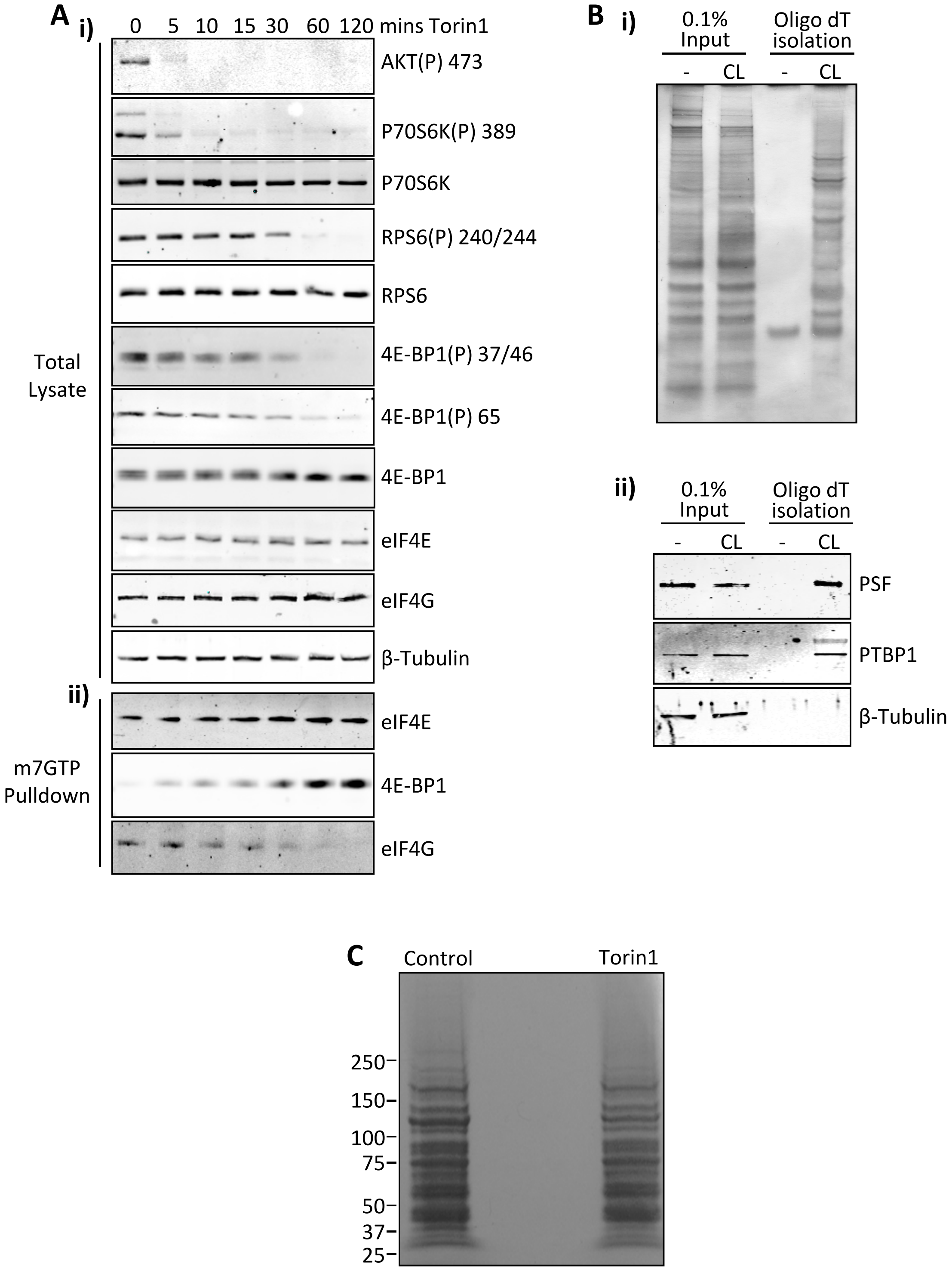

Supplementary Figure S2.

A

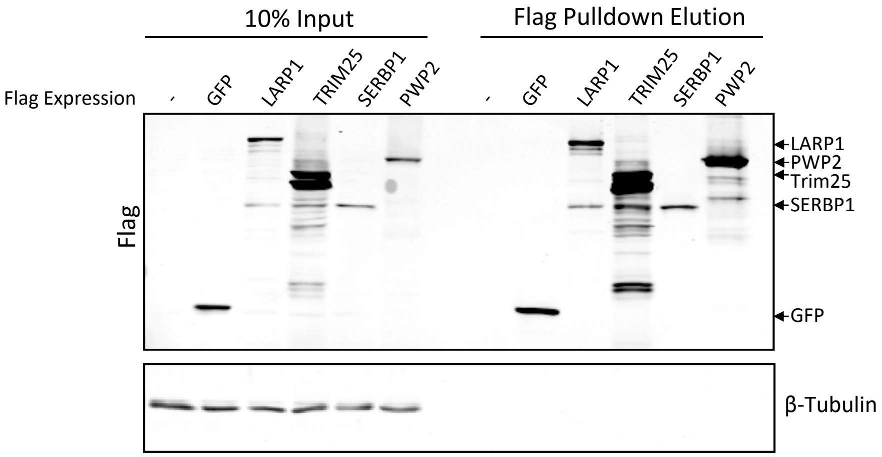

B

RNA binding Protein	Significantly enriched mRNAs
LARP1	2546
TRIM25	0
SERBP1	1261
PWP2	0

C

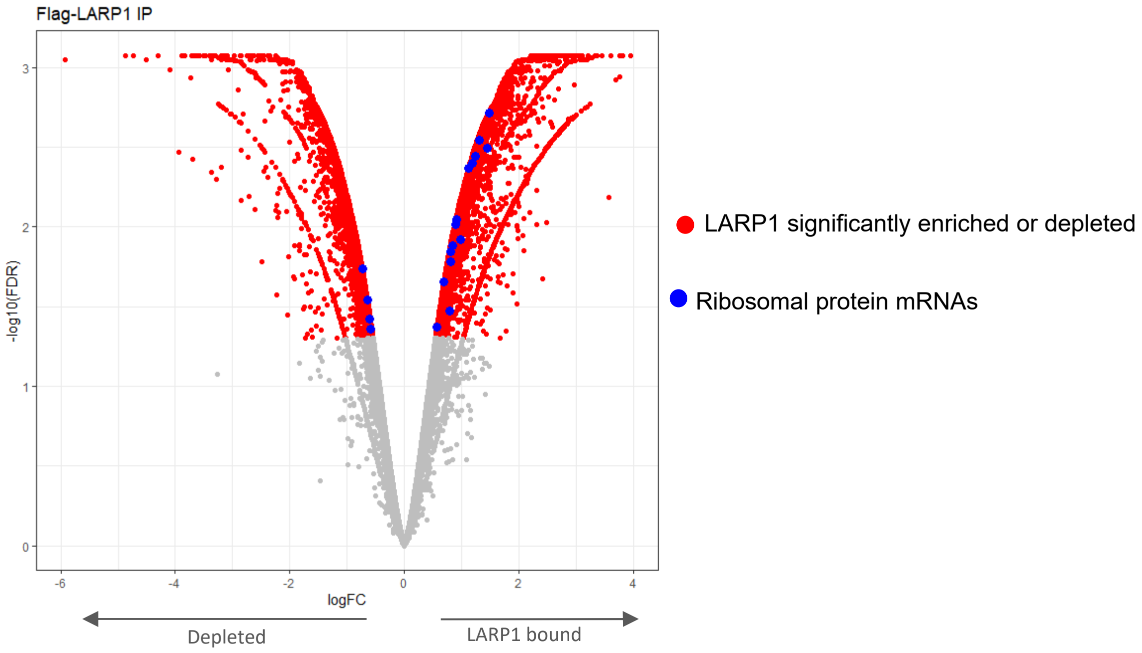

Supplementary Figure S3.

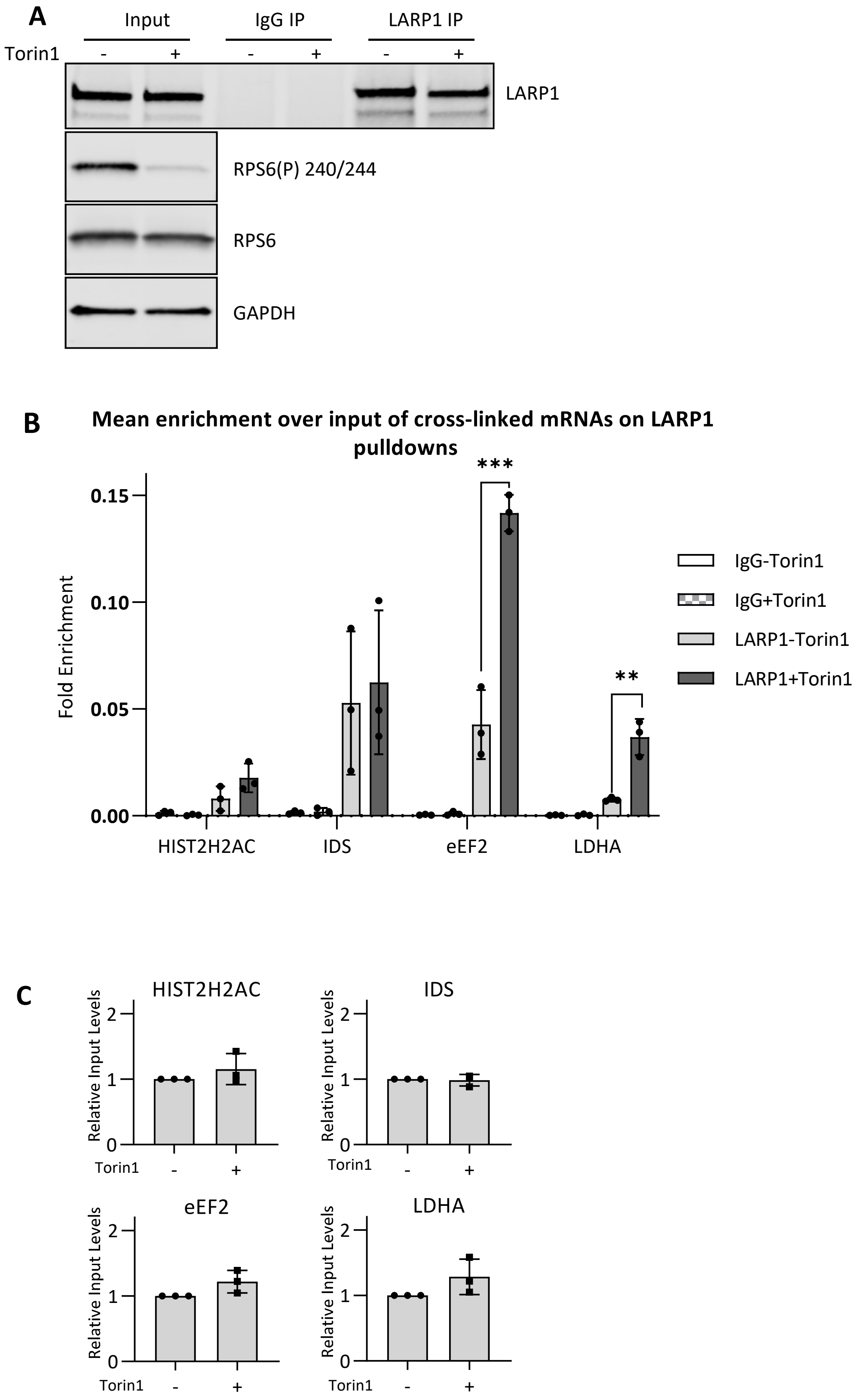

Supplementary Figure S4.

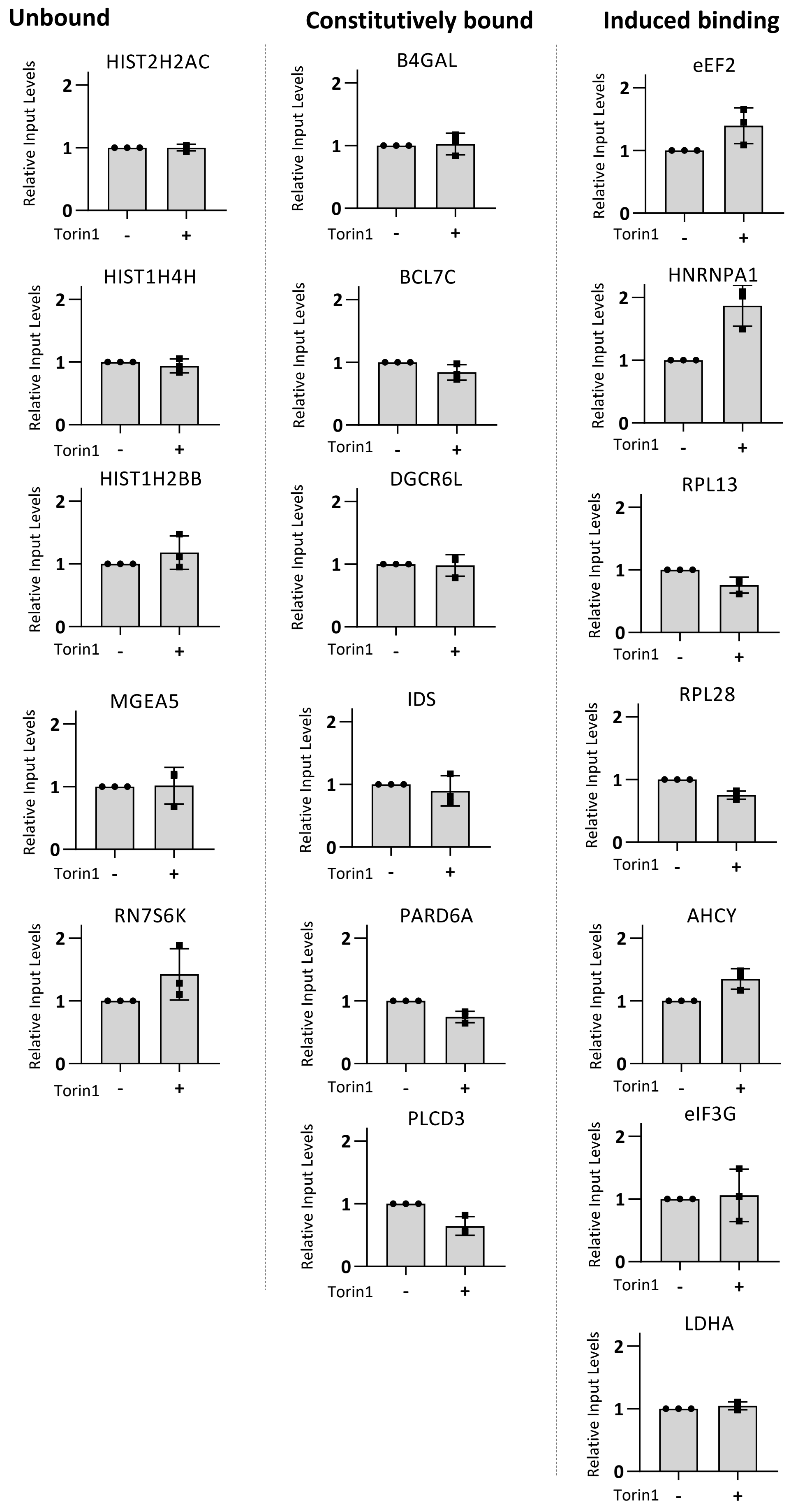

Supplementary Figure S5.

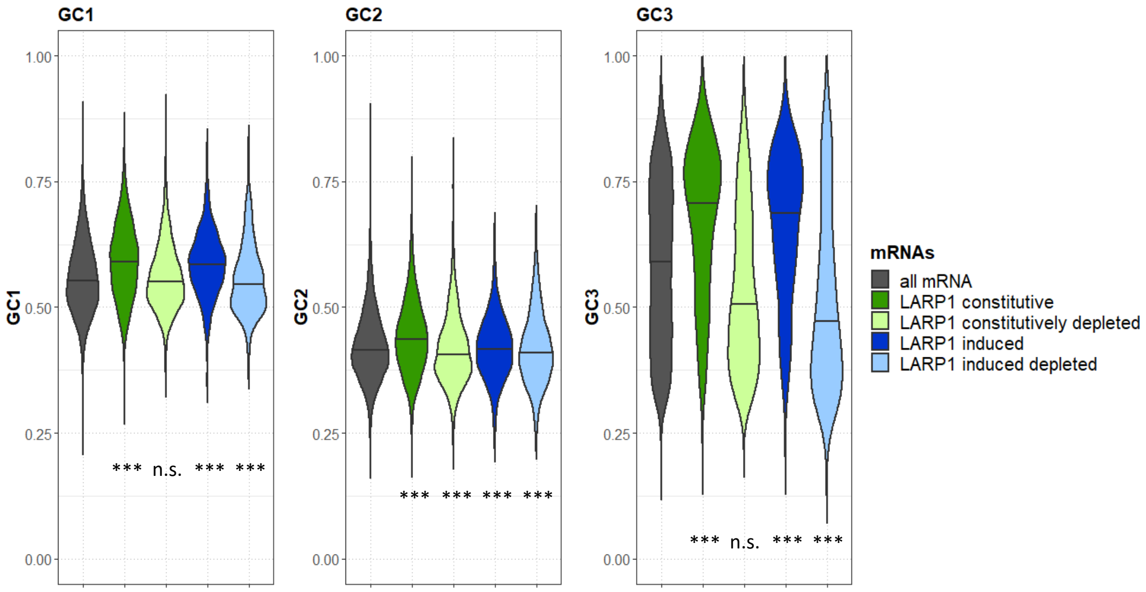

Supplementary Figure S6.

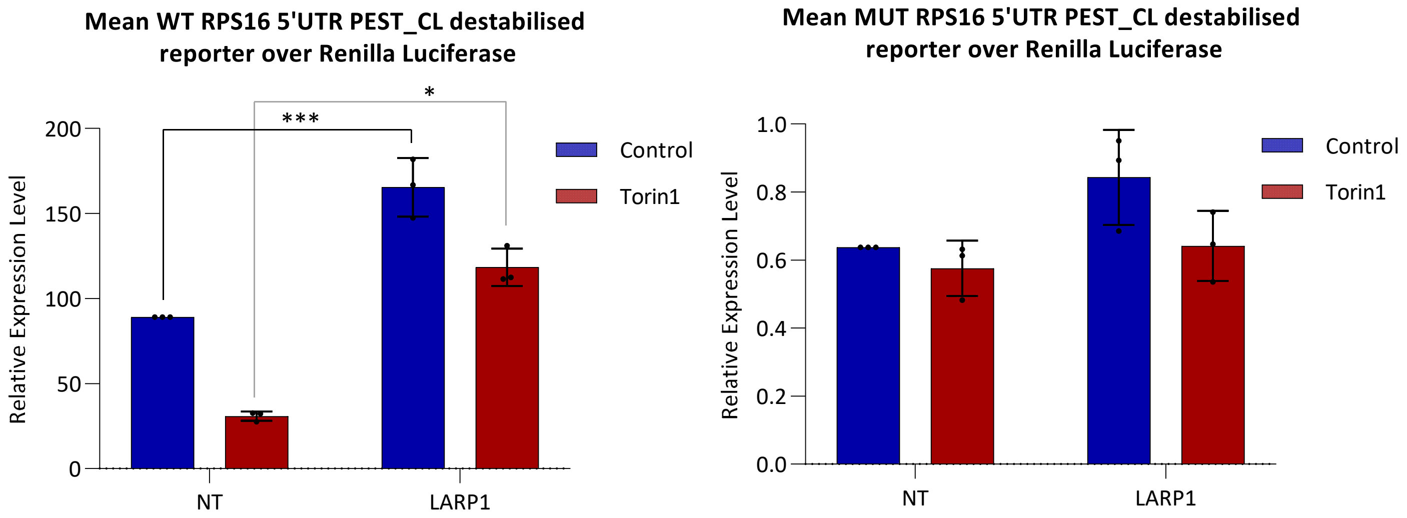

Supplementary Figure S7.

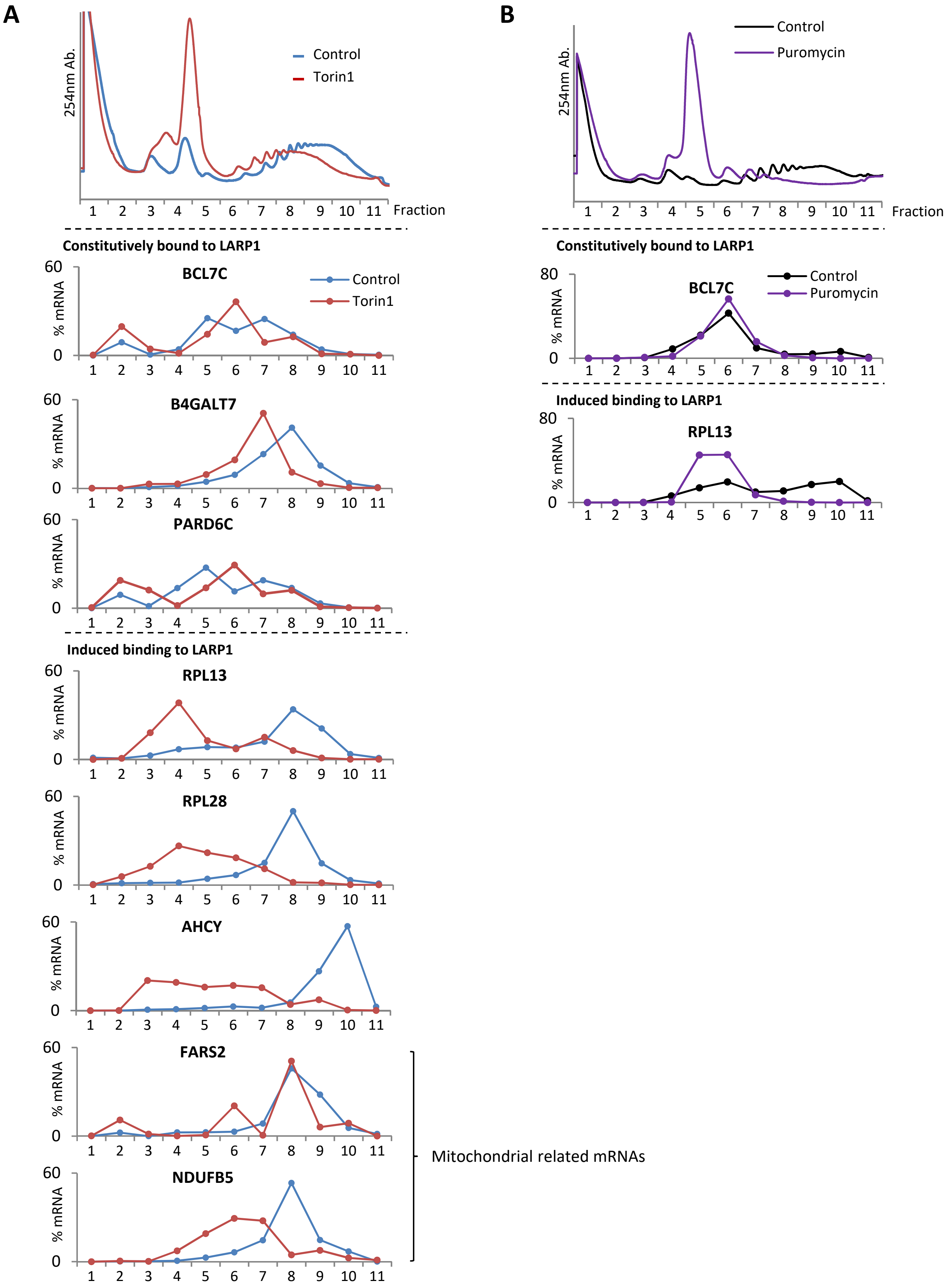

Supplementary Figure S8.

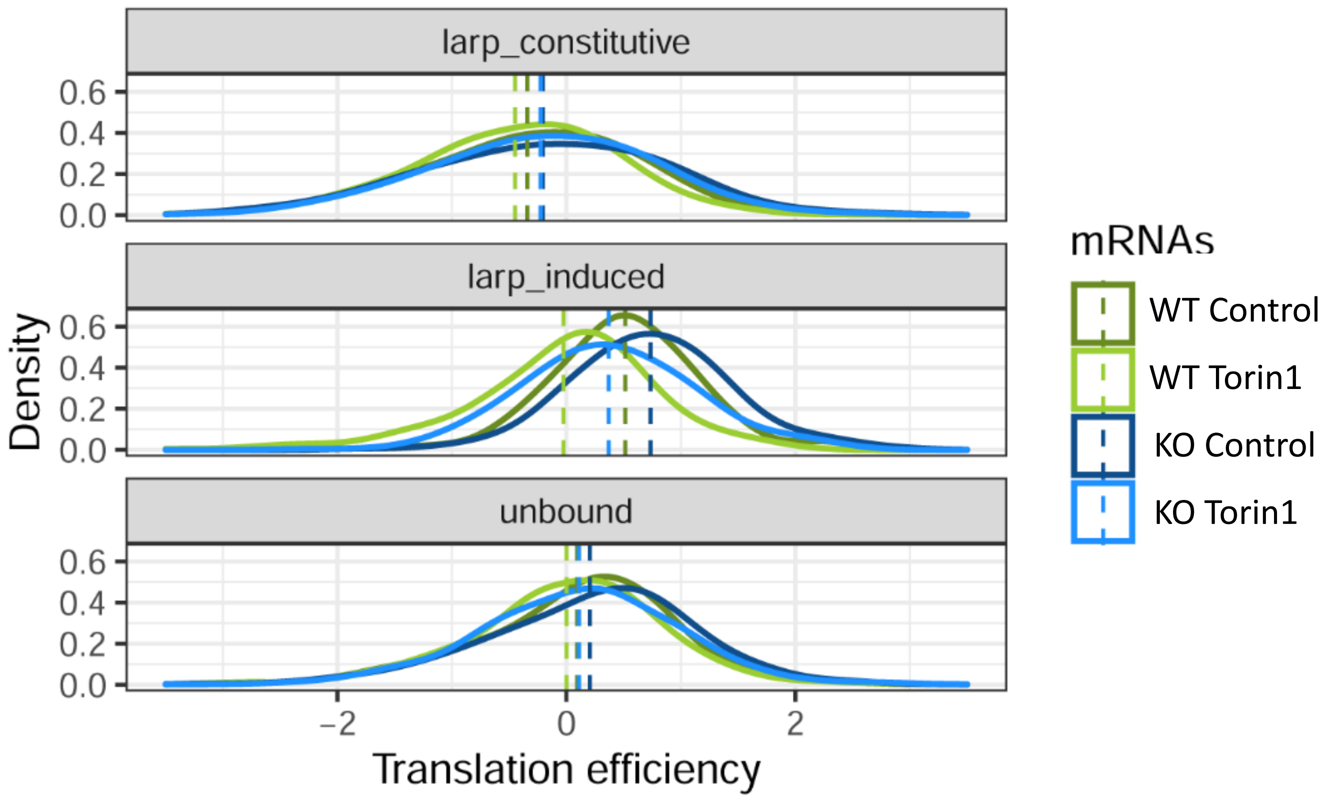

Supplementary Figure S9.

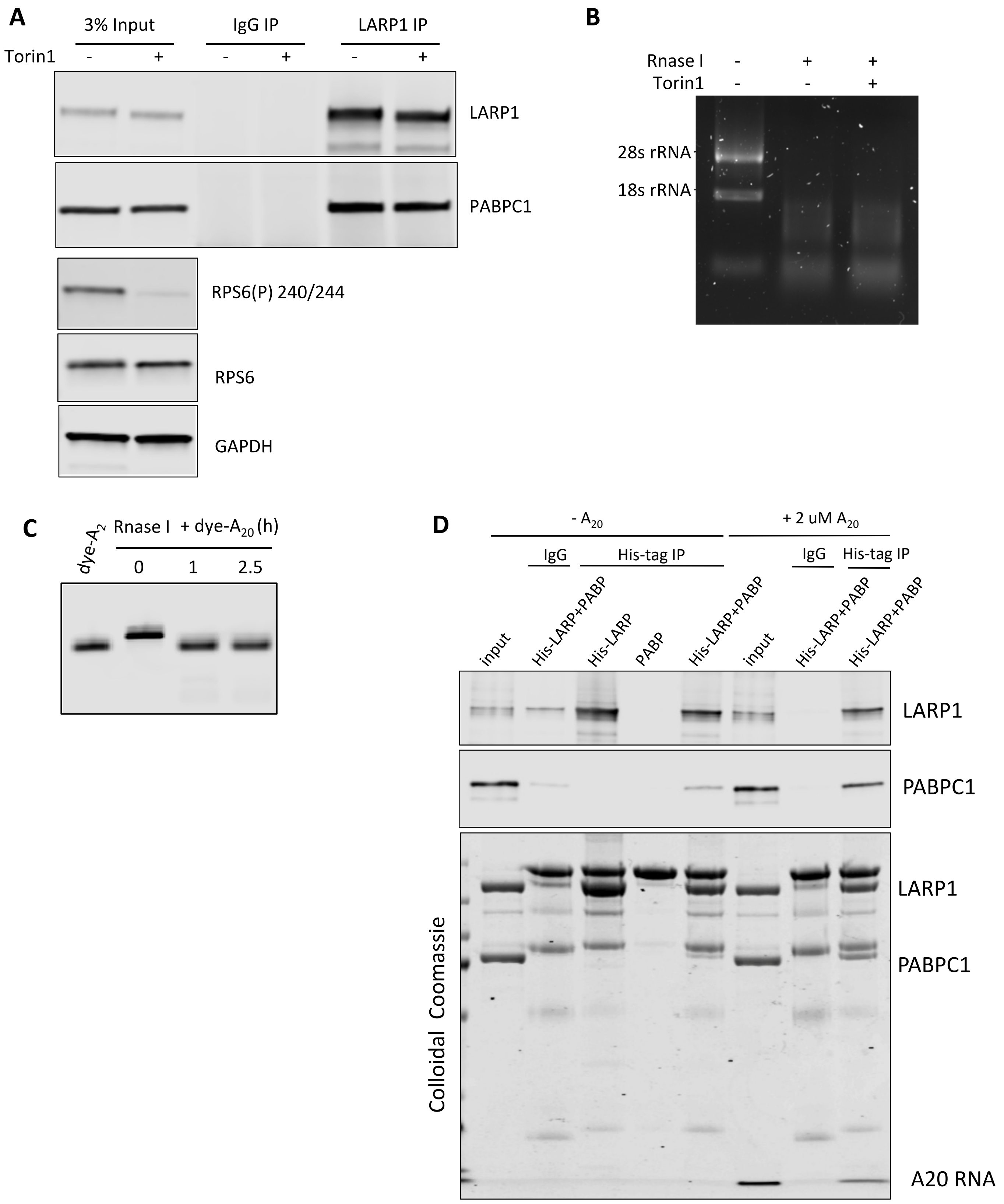

Supplementary Figure S10.

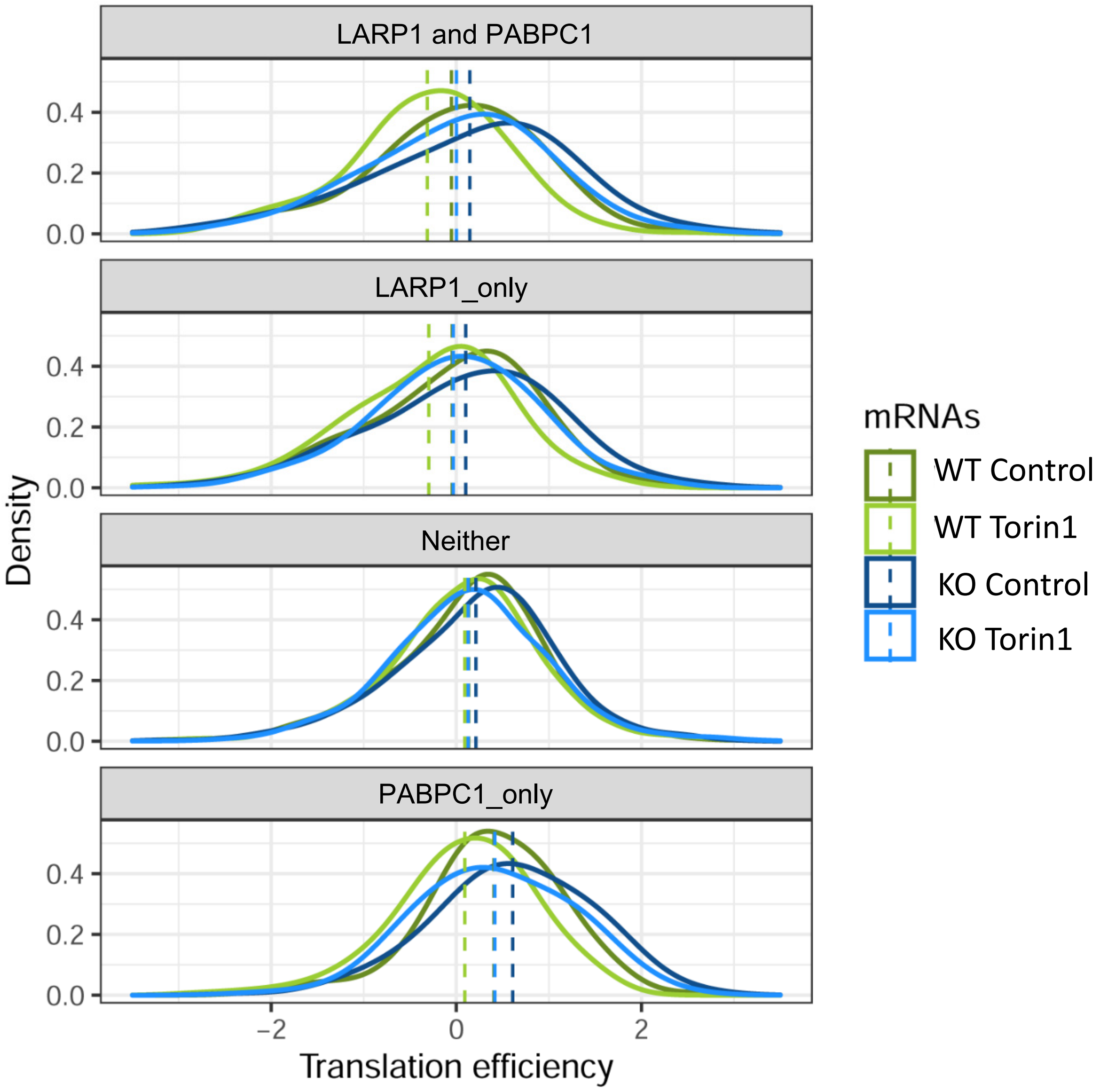

Supplementary Figure S11.

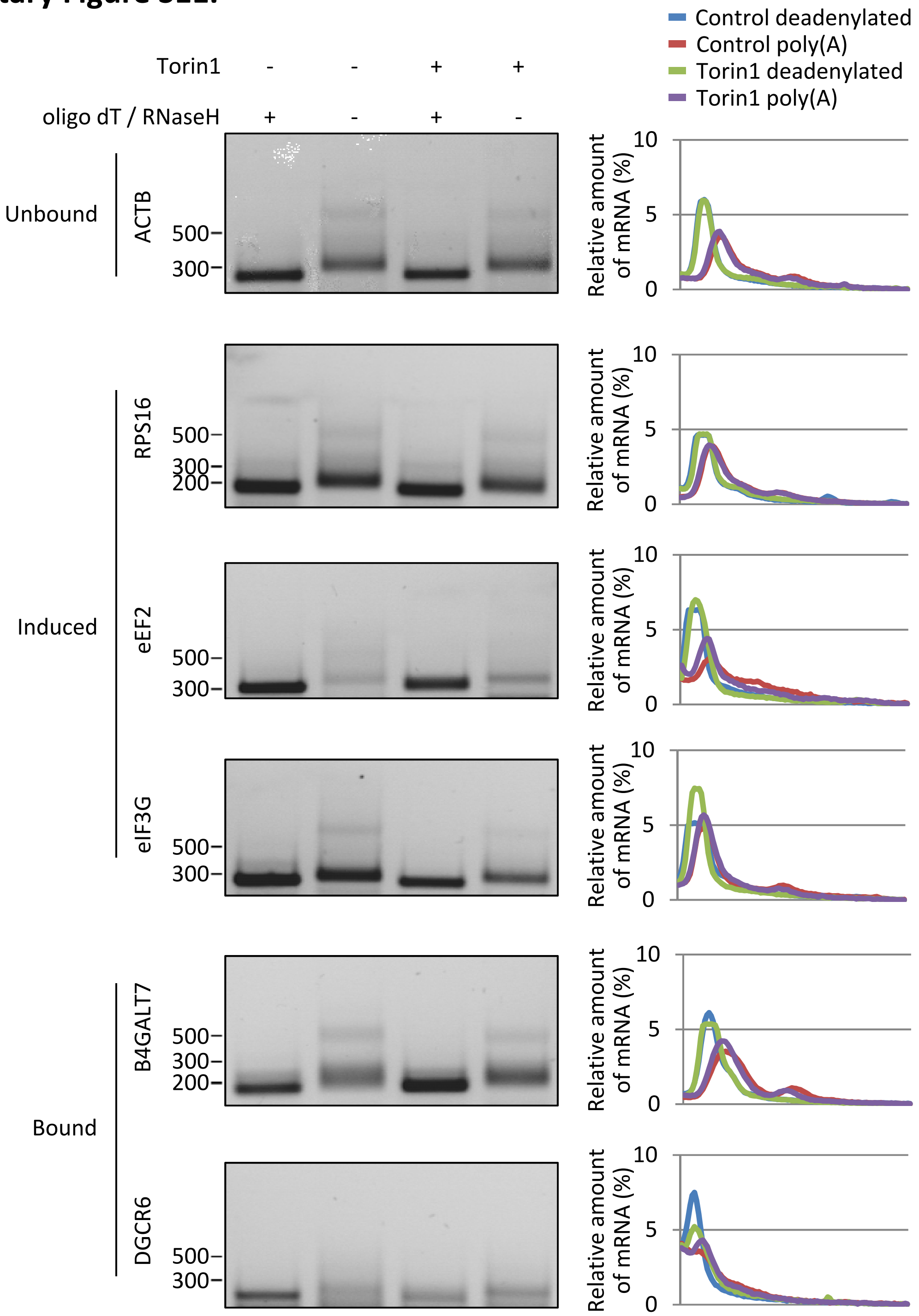

Supplementary Figure S12.

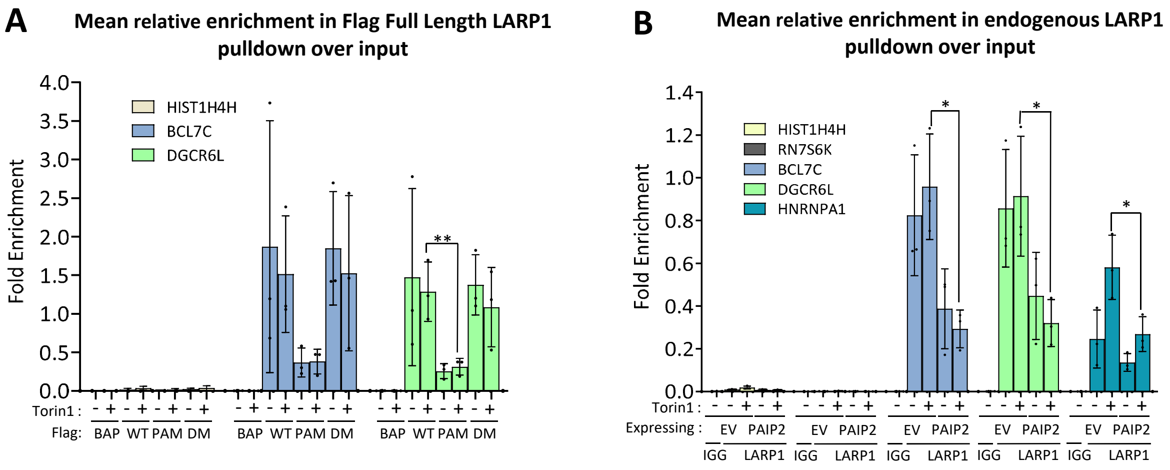

Supplementary Figure S13.

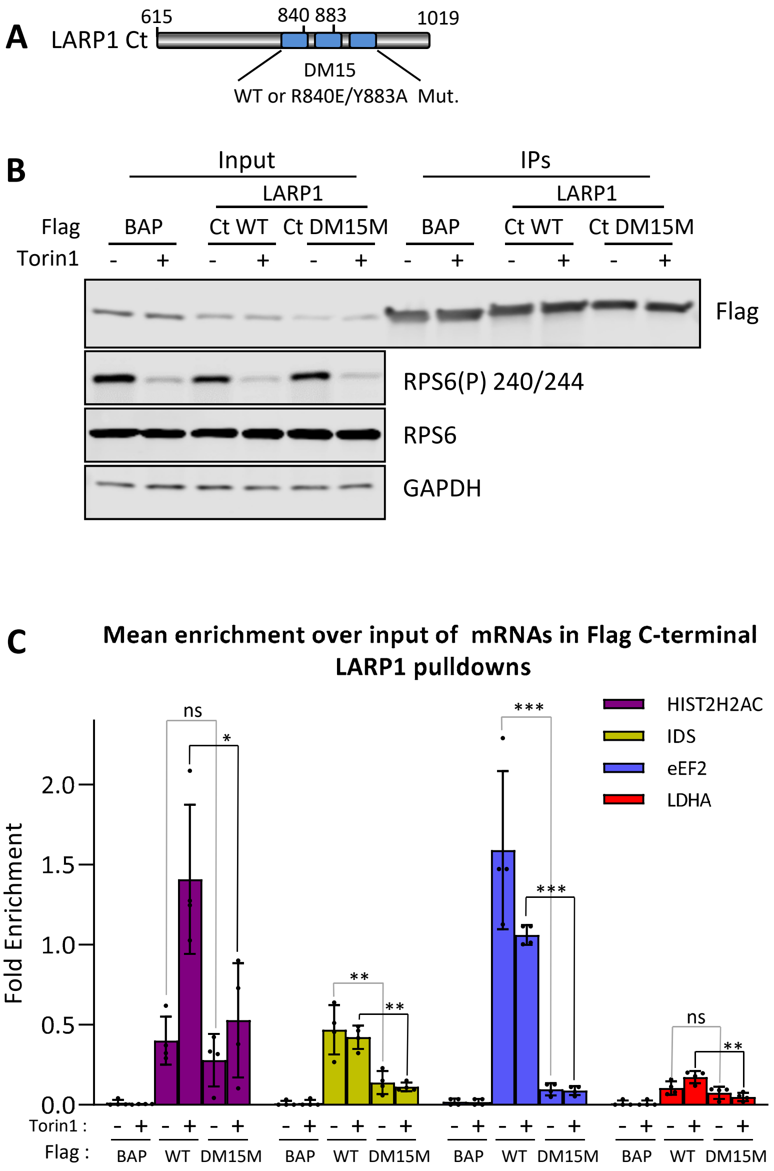

Supplementary Figure S14.

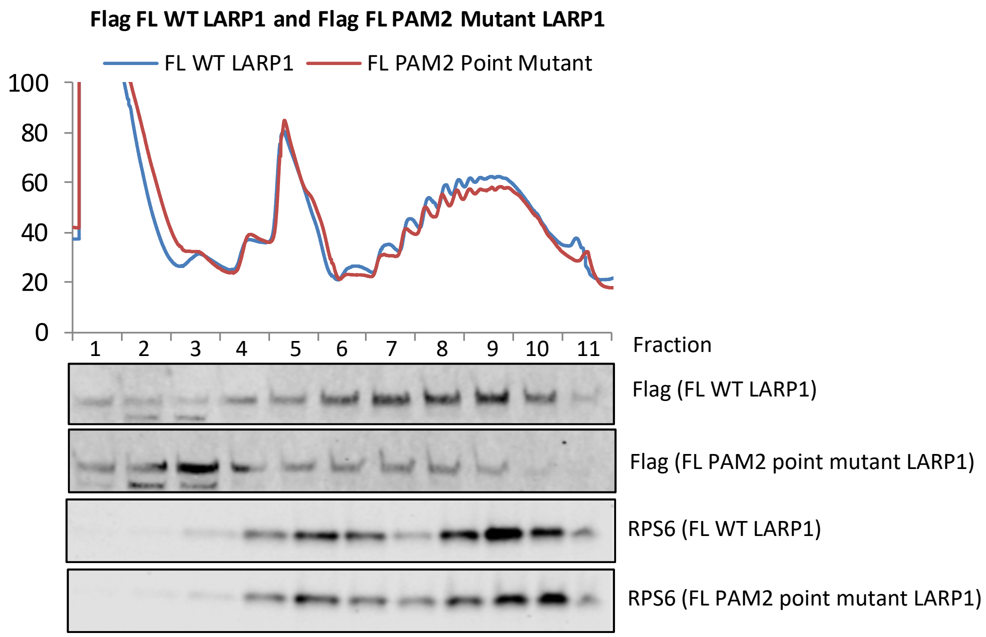

Supplementary Figure S15.

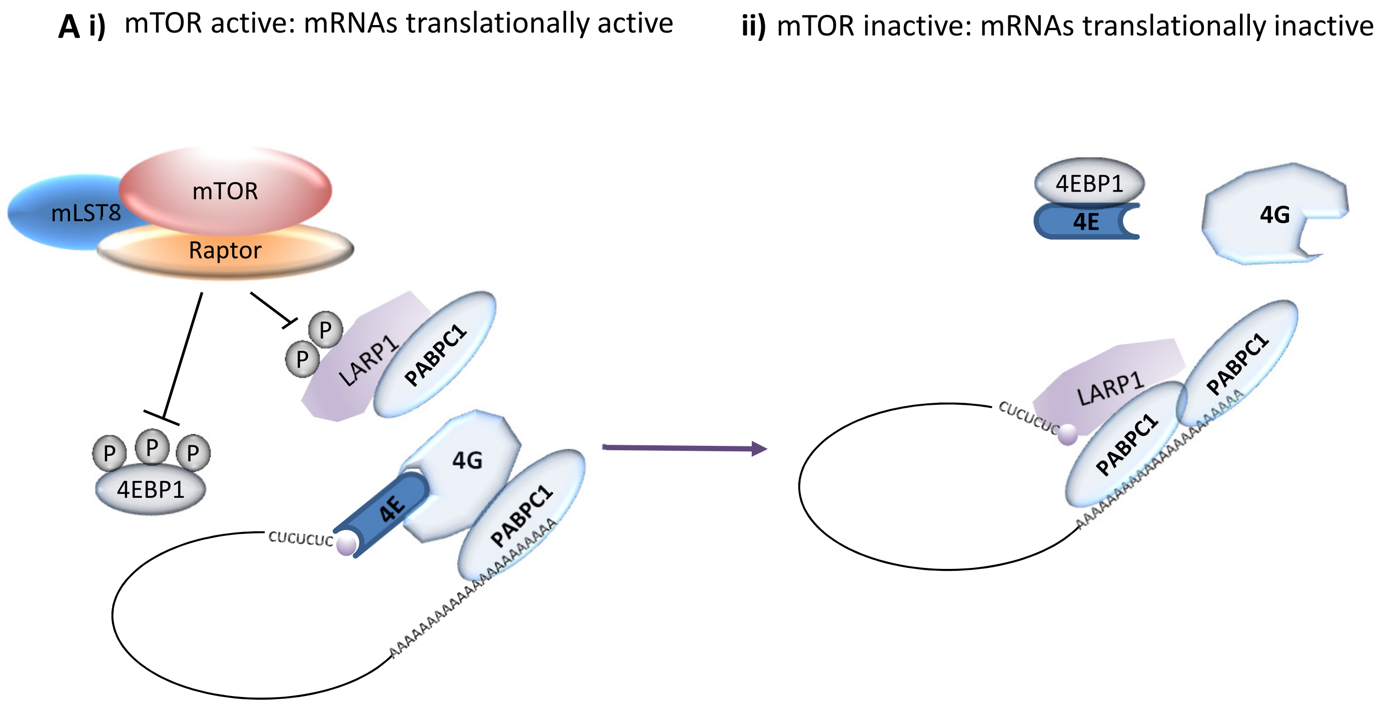

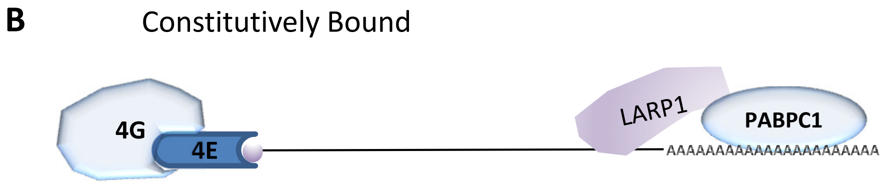

Supplementary Table S2.

RNA Binding Protein	logFC	P.Value	adj.P.Value
TRIM25	0.81043	0.0197397	0.1952342
LARP1	0.610328	0.007865	0.1628654
RPS2	-0.52172	0.0154555	0.1837486
RPSA	-0.54463	0.0097745	0.1628654
SERBP1	-0.56122	0.0126083	0.179879
DDX21	-0.64031	0.0152743	0.1837486
RSL1D1	-0.67644	0.0071327	0.1628654
RPS14	-0.71546	0.0200708	0.1952342
PDCD11	-0.77218	0.003528	0.1628654
PABPC3	-0.81427	0.0067552	0.1628654
NPM1	-0.81734	0.0092363	0.1628654
RPS26	-0.84318	0.0063905	0.1628654
CMYA5	-0.85026	0.0044389	0.1628654
RPS10	-0.86	0.0071637	0.1628654
RPS7	-0.88374	0.0061585	0.1628654
RPS8	-0.89392	0.0178832	0.1913506
PWP2	-0.92622	0.0074484	0.1628654
RPS11	-0.94102	0.0172932	0.1913506
DDX56	-0.97675	0.0151754	0.1837486
EBNA1BP2	-1.04789	0.0070167	0.1628654
RPS18	-1.22268	0.0098937	0.1628654
USP10	-1.44425	0.0125885	0.179879

Increased binding following mTOR inhibition (TRIM25, LARP1)

Decreased binding following mTOR inhibition (RPS2 through USP10)

Supplementary Table S4 - List of qPCR primers and Northern probe-generation primers used in this study.

Gene	Forward Primer (5'-3')	Reverse Primer (5'-3')
**ACHY**	CCCTCAACATGCTTCTGGACGA	AAACTTGCTCTTGGTGACGG
**B4GALT7**	GCTCTCCAAGCAGCACTACC	CGGTAGAACTCGTCGTCCTC
**BCL7C**	TCGGAAGGTTCCCTGCAAAA	GCTCCTCCTTGGTCAGCATT
**DGCR6**	GCTGGCTTCTACGTGACCA	CTGCAGCTTTCGGATGAGT
**eEF2**	CGTGCCATCACTCAACCATAA	CTCCGGACTCTGGAAATAAATATTG
**eIF3G**	CCACCTCATCCTCAACGTC	CTGTCTTCTGTCGCCAAGG
**FARS2**	GGTAGGATTTGCCCAGCAG	GGTAGGATTTGCCCAGCAG
**HIST1H4H**	CCAAACGCAAGACCGTGACA	TGCAGCAAGCAGGAGCCTTA
**HIST2H2AC**	GGCTCGGGACAACAAGAAGA	AGAACGGCCTGGATGTTAGG
**HIST1H2BB**	ACTGCTGCCCTGACTTCTG	AGACTGGATAGATACGACCTCCA
**HNRNPA1**	GCCGAAGAAGCATCGTTAAA	GCTCAACCCTCCAATGAAGA
**IDS**	CCAACTCGACCACAGATGCT	GCCAGTGAGGAAAGAAACGC
**LDHA**	GCAGATTTGGCAGAGAGTATAATG	GACATCATCCTTTATTCCGTAAAGA
**MGEA5**	TGGTCTAGCAGGAGAGTTCCA	AAACTTTGGAGGTAGGAGTCAGTG
**NDUFB5**	ATTTGGGGGCTTCCTCACT	AGTCTTTTCCCATGGTCTCCA
**PARD6A**	GGCTATACGGATGCTCATGG	CCGCTGGAGTCAGCTTCT
**PLCD3**	GCAAGATGAGCTTCAAGGAGA	CGTTGTTGGAGTGGTCACA
**RN7SK**	GGGTTGATTCGGCTGATCT	GGGGATGGTCGTCCTCTT
**RNU4ATAC**	CCATCCTTTTCTTGGGGTTGC	AGCAAAAGCTCTAGTTGATGCG
**RPL13**	CAGCGGCTGAAGGAGTACC	GGTGGCCAGTTTCAGTTCTT
**RPL28**	AAGGTGTCGTGGTGGTCATT	GATGGTGGTCCGCACATAG
**Firefly Luciferase**	CAACTGCATAAGGCTATGAAGAGA	ATTTGTATTCAGCCCATATCGTTT
**B-Actin (N)**	GCGAGCACAGAGCCTCGCCTTT	TGCATCCTGTCGGCAATGCCAG
**eEF2 (N)**	GACAGCGAGGACAAGGACAA	TTGTGCTTGTTGGGGGACTT
**RPS16 (N)**	CAGGTCTTCGGACGCAAGAAG	GTAGGATTTCTGGTAGCGAGCG
**RPL24 (N)**	GTCGAGCTGTGCAGTTTTAGCG	CTGCCTTTGTAGGTGCCTTAGC

Supplementary Table S5. List of primers used for cloning and site-directed mutagenesis

Construct	Primer 5'-3'	Sequence
SERBP1	SERBP1 For	GTATTTTCAGGGCGCCCCTGGGCACTTACAGGAAGGCTTCGGCT
	SERBP1 Rev	GACGGAGCTCGAATTTCAAGCCAGAGCTGGGAATGCCTCTGG GTCA
Trim25	Trim25 For	ACCCAAGCTTGGTACCATGGCAGAGCTGTGCCCCCCTGGCCGA
	Trim25 Rev	AAAATACAGGTTCTCGAGCTTGGGGAGCAGATGGAGAGTGTGGCACCA
PWP2	PWP2 For	GTATTTTCAGGGCGCCAAGTTCGCTTACCGGTTTTCAAATTTGCTGG
	PWP2 Rev	GACGGAGCTCGAATTTCAGGCCAGCATCTCTTCTTCTGAGTCTCTG
Full Length LARP1(2-1019)	HIND3_NTERLARP1_F	CTAAAGCTT CTTTGGAGGGTGCTTTTGTCAAAGAGG
	XBA1_CTERLARP1_R	ATGTCTAGATTACTTTCCCAAAGTCTGTGTGTTCGAGTGC
N-terminal LARP1 (2-598)	HIND3_NTERLARP1_F	CTAAAGCTT CTTTGGAGGGTGCTTTTGTCAAAGAGG
	XBA1_598LARP1_R	ATGTCTAGATTACAGTTCGGCGCTCATCTTGGCA
C-terminal LARP1 (615-1019)	HIND3_615LARP1_F	CTAAAGCTTGCTGAAAAGTTTGAACCTGAGTATTCCCAGATC
	XBA1_CTERLARP1_R	ATGTCTAGATTACTTTCCCAAAGTCTGTGTGTTCGAGTGC
PABPC1	PABPC1_F	TTGGTCTCATGGTAACCCCAGTGCCCCCAGC
	PABPC1_R	AATAGCGGCCGCTTAAACAGTTGGAACACCGGTGGC
LARP1 L423A/F428A	LARP1 L423A/F428A_F	CCCAGCTTGCCAACTGCCCTGAAGCTGTTCCCCGTCAGCACT
	LARP1 L423A/F428A_R	CGGGGAACAGCTTCAGGGCAGTTGGCAAGCTGGGAGAAATCAG
LARP1 R840E	LARP1 R840E_F	GAACACACTCTTCGAGTTCTGGTCCTTCTTCCTCCGA
	LARP1 R840E_R	GAAGGACCAGAACTCGAAGAGTGTGTTCATCTCCTG
LARP1 Y883A	LARP1 Y883A_F	GAAGGCTACAGACGTGGTTTGGAGTGCCTTTTTCG
	LARP1 Y883A_R	GCACTCCAAACCACGTCTGTAGCCTTCTTTGGCGTC
RPS16 MUT	RPS16 MUT_F	GTTGGCCCCGCGCTTCGAGTGACCGGTCGCGGCGCTGCGCGGTG
	RPS16 MUT_R	CAGCGCCGCGACCGGTCACTCGAAGCGCGGGGCCAACCCAGCC

Supplementary Table S6. Polyadenylation Test Primers

General PAT Primers		
**nested anchor**	PAT assay ligation	5rApp/ggtcaccttgatctgaagccagctgtagctatgc/3ddC
**PAT-R0**	PAT assay RT and round 1	GGCATAGCTACAGCTGGC
**PAT-R1**	PAT assay round 2	GCTTCAGATCAAGGTGACCTTTTT
**Target specific primers**		
**Target Gene**	**PAT Round 1 specific primer**	**PAT Round 2 specific primer**
**ACTB**	CCCACTTCTCTCTAAGGAGA	GTGATAGCATTGCTTTCGTGT
**RPS16**	GACATCCGTGTCCGTGTAAA	CATCCTCATCCAGTATGACC
**eEF2**	AGGCAGCAGACACGCCCTC	CACGCCCTCTTAGTAGGGAC
**eIF3G**	CTGCAGGAGCTCTTCCGGC	GCTCCATCTCCCGCATCTAC
**B4GALT7**	GTGCAGAGACACAGTGTAGG	GGTGAGGGTTAGGACTTCAG
**DGCR6**	GCCTGTCACCCTTCATCTGG	AGCCTGCTGCTGTCTGCTTC

Supplementary Table S7. Fold change cut-offs for RNA groups used for subsequent analysis of LARP1 and PABP bound mRNAs

Group	logFC _LARP1_Control	logFC_PABPC1_Control
Depleted in Both	< - 0.5	< - 0.5
LARP1 and PABPC1	> 0.5	> 0.5
LARP1 only	> 0.5	<= 0
PABPC1 only	<=0	> 0.5
